# Supplementary material for: Circulating amino acids and amino acid-related metabolites and risk of breast cancer among predominantly premenopausal women
Source: NPJ Breast Cancer. 2021 May 18;7:54. doi: 10.1038/s41523-021-00262-4 (PMC8131633; doi:10.1038/s41523-021-00262-4)
Supplement: Supplementary file 1 — Supplementary Information [file 41523_2021_262_MOESM1_ESM.pdf]

Supplementary Table 1. : Odds ratios (OR) of breast cancer and 95% confidence intervals (CI) per 1 SD increase for all metabolites in NHSII

Simple model: adjusts for matching factors including menopause status at blood draw, time of blood draw, date/season of blood draw, luteal day at blood draw, fasting status at blood draw, menopausal status at diagnosis and race.

Adjusted model: in addition to matching factors, this model adjusts for BMI at age 18, weight change between age 18 and time of blood draw, age at menarche, parity and age at first birth, family history of breast cancer, personal history of benign breast disease, physical activity, alcohol consumption, exogenous hormone use, breast feeding history.

**Overall BRCA: cases = 1057, controls = 1057**		**Simple Model**			**Adjusted Model**		
**HMDB ID**	**Metabolite**	**OR (95% CI)**	**PVAL**	**QVAL**	**OR (95% CI)**	**PVAL**	**QVAL**
NA	DMGV	0.84 (0.77-0.92)	0.00009	0.012	0.88 (0.79-0.97)	0.011	0.366
NA	2-aminohippuric acid	0.84 (0.76-0.92)	0.00014	0.012	0.85 (0.77-0.93)	0.001	0.052
HMDB29377	piperine	0.85 (0.78-0.93)	0.00046	0.025	0.84 (0.77-0.92)	0.0003	0.037
HMDB00715	kynurenic acid	0.87 (0.79-0.95)	0.003	0.107	0.86 (0.78-0.95)	0.003	0.128
HMDB11394*	C40:7 PE plasmalogen	1.14 (1.05-1.25)	0.003	0.107	1.11 (1.01-1.22)	0.030	0.432
HMDB00168	asparagine	1.14 (1.04-1.26)	0.006	0.166	1.1 (1-1.22)	0.055	0.521
HMDB11211*	C34:3 PC plasmalogen	1.13 (1.03-1.23)	0.008	0.166	1.09 (0.99-1.2)	0.075	0.547
HMDB04824	N2,N2-dimethylguanosine	0.88 (0.81-0.97)	0.008	0.166	0.91 (0.83-1)	0.060	0.530
HMDB10391*	C20:1 LPC	1.13 (1.03-1.25)	0.009	0.166	1.1 (1-1.22)	0.052	0.521
HMDB06344	phenylacetylglutamine	0.89 (0.82-0.97)	0.011	0.191	0.9 (0.82-0.98)	0.022	0.408
HMDB01565	phosphocholine	1.12 (1.02-1.23)	0.018	0.252	1.09 (0.98-1.2)	0.098	0.596
HMDB01855	5-hydroxytryptophol	1.13 (1.02-1.25)	0.018	0.252	1.11 (1-1.24)	0.048	0.521
HMDB00883	valine	0.9 (0.83-0.99)	0.025	0.289	0.94 (0.85-1.03)	0.202	0.596
HMDB04400	5-acetylamino-6-amino-3-methyluracil	0.91 (0.83-0.99)	0.027	0.289	0.92 (0.84-1.01)	0.076	0.547
HMDB10404	C22:6 LPC	1.11 (1.01-1.21)	0.027	0.289	1.06 (0.96-1.17)	0.227	0.603
HMDB11526	C22:6 LPE	1.11 (1.01-1.22)	0.029	0.289	1.08 (0.98-1.2)	0.110	0.596
HMDB32390	2-methyl-4,5-benzoxazole	0.91 (0.83-0.99)	0.030	0.289	0.91 (0.84-1)	0.049	0.521
HMDB01257	spermidine	1.11 (1.01-1.23)	0.036	0.297	1.12 (1.01-1.25)	0.033	0.432
HMDB11103	1,7-dimethyluric acid	0.91 (0.83-0.99)	0.036	0.297	0.92 (0.84-1.01)	0.082	0.547
HMDB00767	pseudouridine	0.91 (0.83-1)	0.039	0.297	0.93 (0.85-1.03)	0.157	0.596
HMDB00064	creatine	1.1 (1-1.21)	0.039	0.297	1.13 (1.02-1.25)	0.015	0.397
HMDB10386*	C18:2 LPC	1.1 (1-1.2)	0.040	0.297	1.05 (0.95-1.16)	0.315	0.603
HMDB00544	5-hydroxymethyl-4-methyluracil	0.9 (0.82-1)	0.043	0.297	0.92 (0.83-1.02)	0.120	0.596
HMDB01276	N1-acetylspermidine	1.1 (1-1.21)	0.043	0.297	1.11 (1.01-1.23)	0.030	0.432
HMDB00687	leucine	0.91 (0.83-1)	0.045	0.302	0.94 (0.86-1.04)	0.246	0.603
HMDB00172	isoleucine	0.91 (0.83-1)	0.049	0.313	0.95 (0.86-1.04)	0.282	0.603
HMDB13325	C10:2 carnitine	0.92 (0.84-1)	0.056	0.341	0.93 (0.84-1.02)	0.103	0.596

HMDB11511	C20:0 LPE	1.09 (1-1.19)	0.057	0.341	1.07 (0.97-1.18)	0.167	0.596
HMDB05923	N4-acetylcytidine	0.92 (0.84-1.01)	0.065	0.367	0.96 (0.87-1.05)	0.387	0.603
HMDB11420*	C38:7 PE plasmalogen	1.09 (0.99-1.19)	0.066	0.367	1.06 (0.97-1.17)	0.199	0.596
HMDB11503	C16:0 LPE	1.09 (0.99-1.19)	0.076	0.373	1.05 (0.96-1.16)	0.280	0.603
HMDB07883*	C34:4 PC	0.92 (0.84-1.01)	0.076	0.373	0.93 (0.85-1.03)	0.157	0.596
NA	C46:2 TAG	0.93 (0.85-1.01)	0.077	0.373	0.94 (0.86-1.03)	0.185	0.596
NA	C36:2 PS plasmalogen	1.08 (0.99-1.19)	0.078	0.373	1.09 (0.99-1.19)	0.083	0.547
HMDB10169	C16:0 SM	1.09 (0.99-1.19)	0.078	0.373	1.07 (0.97-1.18)	0.187	0.596
HMDB10407*	C16:1 LPC plasmalogen	1.08 (0.99-1.19)	0.087	0.403	1.05 (0.96-1.16)	0.285	0.603
HMDB11229*	C38:7 PC plasmalogen	1.08 (0.99-1.18)	0.091	0.407	1.04 (0.94-1.14)	0.467	0.609
HMDB11745	N-acetylmethionine	1.08 (0.99-1.19)	0.097	0.426	1.08 (0.98-1.19)	0.126	0.596
HMDB01431	pyridoxamine	0.92 (0.83-1.02)	0.107	0.450	0.94 (0.85-1.05)	0.281	0.603
HMDB00562	creatinine	0.93 (0.85-1.02)	0.108	0.450	0.92 (0.84-1.01)	0.078	0.547
HMDB00128	guanidinoacetic acid	1.07 (0.98-1.17)	0.113	0.459	1.06 (0.97-1.17)	0.203	0.596
HMDB11507*	C18:2 LPE	1.07 (0.98-1.17)	0.125	0.494	1.05 (0.96-1.15)	0.264	0.603
HMDB01414	putrescine	1.07 (0.98-1.18)	0.133	0.514	1.07 (0.97-1.18)	0.176	0.596
NA	C46:3 TAG	0.94 (0.86-1.02)	0.139	0.524	0.95 (0.87-1.04)	0.309	0.603
NA	C44:2 TAG	0.94 (0.86-1.02)	0.149	0.548	0.95 (0.87-1.04)	0.273	0.603
HMDB00824	C3 carnitine	0.94 (0.86-1.03)	0.166	0.578	0.97 (0.88-1.06)	0.463	0.609
HMDB02000	myristoleic acid	0.94 (0.86-1.03)	0.169	0.578	0.97 (0.89-1.06)	0.523	0.609
HMDB13287	N6,N6-dimethyllysine	0.94 (0.86-1.03)	0.176	0.578	0.94 (0.86-1.03)	0.188	0.596
HMDB06347	C26 carnitine	1.06 (0.97-1.16)	0.178	0.578	1.07 (0.98-1.17)	0.141	0.596
NA	C34:1 DAG_or_TAG_fragment	0.94 (0.86-1.03)	0.179	0.578	0.99 (0.89-1.09)	0.822	0.613
HMDB01563	1-methylguanosine	0.94 (0.86-1.03)	0.186	0.578	0.97 (0.88-1.07)	0.545	0.609
HMDB00631*	glycodeoxycholate/glycochenodeoxycholate	1.06 (0.97-1.16)	0.186	0.578	1.08 (0.98-1.18)	0.123	0.596
HMDB11506*	C18:1 LPE	1.06 (0.97-1.16)	0.186	0.578	1.05 (0.95-1.15)	0.341	0.603
HMDB00259	serotonin	1.06 (0.97-1.16)	0.190	0.578	1.05 (0.96-1.15)	0.300	0.603
HMDB15168	cerulenin	0.94 (0.86-1.03)	0.192	0.578	0.95 (0.86-1.04)	0.258	0.603
HMDB00054	bilirubin	0.94 (0.86-1.03)	0.195	0.578	0.94 (0.85-1.03)	0.178	0.596
HMDB11517	C20:4 LPE	1.06 (0.97-1.16)	0.207	0.605	1.06 (0.97-1.16)	0.198	0.596
HMDB13127	C4-OH carnitine	1.06 (0.97-1.16)	0.218	0.623	1.13 (1.02-1.24)	0.019	0.408
HMDB01906*	aminoisobutyric acid	1.06 (0.97-1.16)	0.226	0.637	1.04 (0.95-1.15)	0.374	0.603
HMDB10384	C18:0 LPC	1.06 (0.97-1.15)	0.237	0.656	1.05 (0.95-1.15)	0.347	0.603
HMDB00187	serine	1.05 (0.96-1.16)	0.260	0.695	1.05 (0.95-1.15)	0.340	0.603
HMDB02013	C4 carnitine	0.95 (0.87-1.04)	0.267	0.695	0.96 (0.87-1.05)	0.319	0.603

HMDB00206	N6-acetyllysine	0.95 (0.87-1.04)	0.280	0.695	0.95 (0.86-1.05)	0.316	0.603
HMDB00688	C5 carnitine	0.95 (0.87-1.04)	0.282	0.695	0.98 (0.9-1.08)	0.754	0.609
HMDB00714	hippurate	1.05 (0.96-1.14)	0.284	0.695	1.02 (0.94-1.12)	0.612	0.609
HMDB01539	ADMA	0.95 (0.86-1.05)	0.293	0.695	0.95 (0.86-1.06)	0.356	0.603
HMDB00705	C6 carnitine	0.95 (0.87-1.04)	0.299	0.695	0.98 (0.89-1.08)	0.648	0.609
HMDB02802	cortisone	0.95 (0.87-1.05)	0.302	0.695	0.96 (0.87-1.05)	0.362	0.603
HMDB00182	lysine	0.95 (0.86-1.05)	0.302	0.695	0.97 (0.87-1.08)	0.554	0.609
HMDB00289	urate	0.95 (0.87-1.05)	0.313	0.695	0.97 (0.89-1.07)	0.579	0.609
HMDB01396	glycocholate	1.05 (0.96-1.14)	0.314	0.695	1.06 (0.96-1.16)	0.231	0.603
HMDB04193	N1-methyl-2-pyridone-5-carboxamide	0.96 (0.87-1.05)	0.319	0.695	0.97 (0.88-1.06)	0.475	0.609
HMDB00725	hydroxyproline	0.96 (0.88-1.04)	0.321	0.695	0.96 (0.88-1.05)	0.413	0.609
HMDB02815*	C18:1 LPC	1.05 (0.96-1.14)	0.327	0.695	1.01 (0.91-1.11)	0.905	0.613
HMDB00210	pantothenate	1.04 (0.96-1.14)	0.335	0.695	1.05 (0.95-1.15)	0.346	0.603
HMDB00062	carnitine	0.96 (0.87-1.05)	0.337	0.695	0.97 (0.88-1.07)	0.527	0.609
HMDB03681	4-acetamidobutanoate	0.96 (0.88-1.05)	0.344	0.695	0.96 (0.87-1.05)	0.372	0.603
HMDB00033	carnosine	0.96 (0.88-1.05)	0.344	0.695	0.96 (0.87-1.05)	0.366	0.603
HMDB00043	betaine	1.05 (0.95-1.15)	0.352	0.695	1.04 (0.94-1.15)	0.441	0.609
HMDB01008	biliverdin	1.04 (0.95-1.14)	0.355	0.695	1.04 (0.95-1.14)	0.427	0.609
NA	C34:3 DAG_or_TAG_fragment	0.96 (0.88-1.05)	0.355	0.695	1.02 (0.92-1.12)	0.762	0.609
NA	threosphingosine	0.95 (0.86-1.05)	0.358	0.695	0.98 (0.88-1.09)	0.704	0.609
HMDB04827	proline betaine	0.96 (0.88-1.05)	0.362	0.695	0.94 (0.86-1.02)	0.149	0.596
NA	C34:2 DAG_or_TAG_fragment	0.96 (0.88-1.05)	0.367	0.695	1.02 (0.92-1.13)	0.678	0.609
HMDB00812	N-acetylaspartic acid	1.04 (0.95-1.14)	0.370	0.695	1.04 (0.95-1.15)	0.397	0.609
HMDB13326	C12:1 carnitine	0.96 (0.88-1.05)	0.371	0.695	0.98 (0.89-1.08)	0.710	0.609
HMDB04030	21-deoxycortisol	0.96 (0.88-1.05)	0.372	0.695	0.95 (0.86-1.04)	0.293	0.603
HMDB00161	alanine	0.96 (0.87-1.05)	0.373	0.695	0.99 (0.9-1.1)	0.862	0.613
HMDB04620	N-alpha-acetylarginine	0.96 (0.88-1.05)	0.374	0.695	0.96 (0.88-1.06)	0.424	0.609
HMDB00791	C8 carnitine	0.96 (0.88-1.05)	0.379	0.695	0.97 (0.89-1.07)	0.586	0.609
HMDB00289	uric acid	0.96 (0.88-1.05)	0.387	0.695	0.98 (0.89-1.08)	0.699	0.609
HMDB04620	N-alpha-acetyarginine	0.96 (0.88-1.05)	0.387	0.695	0.96 (0.88-1.06)	0.429	0.609
HMDB08952*	C34:2 PE plasmalogen	1.04 (0.95-1.13)	0.396	0.695	1.02 (0.93-1.11)	0.715	0.609
HMDB00897	7-methylguanine	0.96 (0.87-1.05)	0.396	0.695	0.96 (0.87-1.06)	0.461	0.609
HMDB00630	cytosine	0.96 (0.88-1.05)	0.401	0.695	0.96 (0.87-1.05)	0.340	0.603
HMDB34169	methyl N-methylanthranilate	1.04 (0.95-1.14)	0.401	0.695	1.04 (0.94-1.14)	0.448	0.609
HMDB00651	C10 carnitine	0.96 (0.88-1.05)	0.407	0.697	0.97 (0.88-1.07)	0.541	0.609

HMDB15150	sulfamethoxazole	1.05 (0.93-1.19)	0.414	0.697	1.05 (0.92-1.19)	0.468	0.609
HMDB00092	dimethylglycine	1.04 (0.95-1.13)	0.420	0.697	1.06 (0.97-1.17)	0.197	0.596
HMDB00026	N-carbamoyl-beta-alanine	0.96 (0.88-1.05)	0.428	0.697	0.96 (0.87-1.05)	0.359	0.603
HMDB11441*	C36:3 PE plasmalogen	1.04 (0.95-1.13)	0.428	0.697	1.01 (0.92-1.11)	0.776	0.609
HMDB13130	C5-DC carnitine	0.97 (0.88-1.05)	0.431	0.697	0.97 (0.88-1.06)	0.474	0.609
NA	C36:2 DAG_or_TAG_fragment	0.96 (0.88-1.06)	0.439	0.697	1.03 (0.93-1.13)	0.589	0.609
HMDB59824	4-hydroxy-3-methylacetophenone	0.96 (0.88-1.06)	0.443	0.697	0.99 (0.9-1.1)	0.875	0.613
HMDB10382	C16:0 LPC	1.04 (0.95-1.13)	0.444	0.697	1.02 (0.93-1.12)	0.662	0.609
HMDB13678	4-hydroxyhippurate	1.03 (0.95-1.13)	0.450	0.697	1.04 (0.95-1.14)	0.375	0.603
HMDB11410*	C36:5 PE plasmalogen	1.03 (0.95-1.13)	0.450	0.697	1.04 (0.95-1.14)	0.381	0.603
HMDB01847	caffeine	0.97 (0.88-1.06)	0.461	0.697	0.98 (0.89-1.07)	0.632	0.609
HMDB01325	N6,N6,N6-trimethyllysine	0.97 (0.88-1.06)	0.465	0.697	0.97 (0.88-1.06)	0.490	0.609
HMDB10383*	C16:1 LPC	0.97 (0.88-1.06)	0.466	0.697	0.97 (0.89-1.07)	0.593	0.609
HMDB09003*	C38:4 PE	0.97 (0.89-1.06)	0.468	0.697	1 (0.91-1.1)	0.944	0.613
HMDB00630*	cytosine_isomer	1.03 (0.94-1.13)	0.470	0.697	1.01 (0.92-1.12)	0.775	0.609
HMDB13331	C14:2 carnitine	0.97 (0.89-1.06)	0.481	0.697	0.98 (0.89-1.07)	0.643	0.609
HMDB00991*	2-aminooctanoic acid	1.03 (0.95-1.13)	0.482	0.697	1.02 (0.93-1.12)	0.635	0.609
HMDB00641	glutamine	1.03 (0.94-1.13)	0.485	0.697	0.99 (0.9-1.09)	0.867	0.613
HMDB11253*	C38:5 PE plasmalogen	1.03 (0.95-1.12)	0.493	0.697	1.04 (0.95-1.14)	0.355	0.603
HMDB00133	guanosine	0.97 (0.89-1.06)	0.501	0.697	0.98 (0.89-1.07)	0.600	0.609
HMDB05862	2-methylguanosine	1.03 (0.94-1.14)	0.502	0.697	1.05 (0.95-1.16)	0.345	0.603
HMDB01859	acetaminophen	0.97 (0.89-1.06)	0.503	0.697	0.95 (0.87-1.04)	0.269	0.603
HMDB01348	C18:0 SM	1.03 (0.94-1.14)	0.503	0.697	1.07 (0.97-1.19)	0.193	0.596
NA	glutamic acid amide	1.03 (0.94-1.13)	0.515	0.703	0.99 (0.9-1.09)	0.831	0.613
HMDB09102*	C38:6 PE	1.03 (0.94-1.13)	0.519	0.703	1.02 (0.93-1.12)	0.692	0.609
NA	N-methylproline	0.97 (0.89-1.06)	0.526	0.703	0.97 (0.88-1.06)	0.455	0.609
HMDB02250	C12 carnitine	0.97 (0.89-1.06)	0.527	0.703	0.99 (0.9-1.08)	0.754	0.609
HMDB02064	N-acetylputrescine	0.97 (0.89-1.06)	0.528	0.703	0.98 (0.89-1.07)	0.638	0.609
HMDB00063	cortisol	0.97 (0.88-1.07)	0.545	0.706	0.96 (0.87-1.06)	0.450	0.609
NA	C36:3 PS plasmalogen	0.97 (0.89-1.06)	0.546	0.706	0.99 (0.9-1.08)	0.744	0.609
HMDB00991*	2-aminooctanoic acid	0.97 (0.87-1.08)	0.551	0.706	0.97 (0.87-1.08)	0.536	0.609
NA	C16:1 SM	0.97 (0.88-1.07)	0.553	0.706	1.01 (0.91-1.12)	0.849	0.613
HMDB00898	1-methylhistamine	1.03 (0.94-1.12)	0.558	0.706	1.01 (0.92-1.11)	0.768	0.609
HMDB12103	C22:0 SM	0.97 (0.87-1.08)	0.559	0.706	0.99 (0.89-1.11)	0.920	0.613
HMDB00853	N-acetylgalactosamine	1.03 (0.93-1.13)	0.567	0.706	1.06 (0.96-1.17)	0.259	0.603

HMDB07869*	C30:0 PC	0.98 (0.89-1.06)	0.568	0.706	0.96 (0.88-1.05)	0.383	0.603
NA	C38:5 DAG_or_TAG_fragment	0.97 (0.89-1.07)	0.570	0.706	1.02 (0.93-1.13)	0.649	0.609
HMDB10395	C20:4 LPC	1.02 (0.94-1.12)	0.590	0.727	1.01 (0.92-1.11)	0.817	0.613
HMDB00462	allantoin	0.97 (0.89-1.07)	0.595	0.728	1.01 (0.91-1.11)	0.903	0.613
HMDB11220*	C36:5 PC plasmalogen	1.02 (0.94-1.12)	0.607	0.731	1.01 (0.92-1.11)	0.769	0.609
HMDB12097	C14:0 SM	0.98 (0.89-1.07)	0.608	0.731	0.98 (0.89-1.08)	0.643	0.609
HMDB09012*	C40:6 PE	0.98 (0.89-1.07)	0.614	0.731	0.99 (0.9-1.09)	0.864	0.613
HMDB05066	C14 carnitine	1.02 (0.94-1.12)	0.616	0.731	1.04 (0.95-1.14)	0.360	0.603
HMDB00301	urocanic acid	0.98 (0.88-1.08)	0.621	0.732	1 (0.9-1.1)	0.931	0.613
HMDB07874*	C32:2 PC	0.98 (0.9-1.07)	0.631	0.738	0.99 (0.9-1.09)	0.839	0.613
HMDB10403	C22:5 LPC	0.98 (0.9-1.07)	0.649	0.740	0.95 (0.87-1.05)	0.316	0.603
HMDB00696	methionine	1.02 (0.93-1.12)	0.654	0.740	1.01 (0.92-1.12)	0.813	0.613
HMDB03334	SDMA	0.98 (0.88-1.08)	0.657	0.740	0.96 (0.86-1.07)	0.413	0.609
HMDB05015	gabapentin	1.04 (0.88-1.23)	0.658	0.740	1.03 (0.87-1.23)	0.708	0.609
HMDB10379	C14:0 LPC	0.98 (0.9-1.07)	0.661	0.740	0.99 (0.9-1.08)	0.799	0.613
HMDB11343*	C34:3 PE plasmalogen	1.02 (0.93-1.11)	0.664	0.740	1 (0.92-1.1)	0.928	0.613
HMDB11478*	C18:3 LPE	1.02 (0.93-1.11)	0.672	0.740	0.99 (0.91-1.09)	0.905	0.613
HMDB02014	C14:1 carnitine	0.98 (0.9-1.07)	0.673	0.740	1 (0.91-1.09)	0.931	0.613
HMDB08994*	C36:2 PE	0.98 (0.9-1.07)	0.673	0.740	1.01 (0.92-1.11)	0.876	0.613
HMDB00679	homocitrulline	0.98 (0.89-1.08)	0.676	0.740	0.97 (0.88-1.07)	0.546	0.609
HMDB00719	homoserine	1.02 (0.92-1.13)	0.686	0.743	1.02 (0.92-1.14)	0.694	0.609
HMDB00716	pipecolic acid	0.98 (0.89-1.08)	0.689	0.743	0.99 (0.89-1.09)	0.765	0.609
HMDB00158	tyrosine	0.98 (0.9-1.08)	0.696	0.744	1.01 (0.91-1.11)	0.892	0.613
Internal Standard	valine-d8	0.98 (0.88-1.09)	0.702	0.744	0.98 (0.88-1.1)	0.760	0.609
HMDB08928*	C34:2 PE	0.98 (0.9-1.08)	0.716	0.744	1 (0.91-1.1)	0.958	0.619
HMDB04207	alpha-glutamyllysine	1.02 (0.92-1.13)	0.720	0.744	1.03 (0.92-1.15)	0.623	0.609
HMDB04953*	C24:1 Ceramide (d18:1)	0.98 (0.9-1.08)	0.725	0.744	0.99 (0.9-1.09)	0.854	0.613
HMDB01476	3-hydroxyanthranilic acid	1.02 (0.93-1.12)	0.727	0.744	1.03 (0.93-1.13)	0.581	0.609
HMDB03282	1-methylguanine	0.98 (0.9-1.08)	0.736	0.744	1 (0.91-1.1)	0.941	0.613
HMDB13713	N-acetyltryptophan	0.99 (0.91-1.07)	0.737	0.744	0.96 (0.88-1.05)	0.419	0.609
HMDB08937*	C36:4 PE	0.98 (0.9-1.08)	0.738	0.744	0.99 (0.9-1.1)	0.897	0.613
HMDB00248	thyroxine	0.98 (0.9-1.08)	0.739	0.744	1.02 (0.92-1.12)	0.753	0.609
HMDB00167	threonine	1.02 (0.92-1.13)	0.740	0.744	1.02 (0.91-1.13)	0.744	0.609
HMDB03357	N-acetylornithine	1.02 (0.93-1.11)	0.743	0.744	0.99 (0.9-1.09)	0.805	0.613
HMDB00001	1-methylhistidine	0.99 (0.9-1.08)	0.748	0.745	0.98 (0.89-1.07)	0.609	0.609

HMDB00201	C2 carnitine	0.99 (0.9-1.08)	0.759	0.750	1.01 (0.92-1.11)	0.798	0.613
HMDB32055	N-acetylhistidine	0.99 (0.9-1.08)	0.762	0.750	0.98 (0.89-1.07)	0.611	0.609
HMDB00925	trimethylamine-N-oxide	1.01 (0.93-1.1)	0.775	0.757	1.04 (0.95-1.14)	0.385	0.603
HMDB02172	diacetylspermine	1.01 (0.93-1.11)	0.783	0.759	1.03 (0.94-1.13)	0.504	0.609
NA	C36:4 DAG_or_TAG_fragment	1.01 (0.93-1.1)	0.785	0.759	1.06 (0.97-1.17)	0.186	0.596
HMDB04949	C16:0 Ceramide (d18:1)	1.01 (0.92-1.11)	0.798	0.766	1.03 (0.93-1.13)	0.585	0.609
HMDB02172	N1,N12-diacetylspermine	1.01 (0.92-1.11)	0.807	0.767	1.03 (0.94-1.13)	0.502	0.609
HMDB29416	NMMA	0.99 (0.89-1.09)	0.808	0.767	1.02 (0.91-1.13)	0.777	0.609
HMDB00479	methylhistidine	0.99 (0.91-1.08)	0.827	0.778	0.98 (0.9-1.08)	0.720	0.609
HMDB02366	C5:1 carnitine	0.99 (0.91-1.08)	0.831	0.778	0.99 (0.9-1.09)	0.843	0.613
NA	C34:1 DAG_or_TAG_fragment	1.01 (0.92-1.1)	0.836	0.778	1.07 (0.97-1.17)	0.191	0.596
HMDB00875	trigonelline	0.99 (0.91-1.08)	0.838	0.778	0.98 (0.9-1.08)	0.742	0.609
HMDB00162	proline	0.99 (0.91-1.08)	0.846	0.778	1 (0.91-1.1)	0.972	0.622
HMDB00159	phenylalanine	1.01 (0.92-1.11)	0.847	0.778	1.03 (0.94-1.14)	0.519	0.609
HMDB00112	GABA	1.01 (0.92-1.1)	0.852	0.778	1.03 (0.94-1.14)	0.492	0.609
HMDB00699	1-methylnicotinamide	0.99 (0.91-1.09)	0.863	0.784	0.97 (0.88-1.07)	0.525	0.609
HMDB13288	C9 carnitine	0.99 (0.91-1.08)	0.869	0.784	0.99 (0.9-1.08)	0.778	0.609
NA	ectoine	1.01 (0.92-1.1)	0.873	0.784	1.01 (0.92-1.1)	0.873	0.613
HMDB13631	oleoyl glycine	1.01 (0.92-1.1)	0.887	0.784	1.01 (0.92-1.12)	0.766	0.609
HMDB00929	tryptophan	1.01 (0.92-1.1)	0.893	0.784	0.99 (0.91-1.09)	0.900	0.613
HMDB11512*	C20:1 LPE	0.99 (0.91-1.09)	0.896	0.784	0.98 (0.89-1.07)	0.648	0.609
HMDB11756	N-acetylleucine	1.01 (0.92-1.1)	0.896	0.784	1.05 (0.95-1.15)	0.349	0.603
Internal Standard	phenylalanine-d8	1.01 (0.9-1.13)	0.901	0.784	1 (0.89-1.12)	0.965	0.620
HMDB10387*	C18:3 LPC	0.99 (0.91-1.09)	0.902	0.784	0.98 (0.89-1.08)	0.740	0.609
HMDB08925*	C34:0 PE	1 (0.92-1.09)	0.914	0.784	0.99 (0.91-1.09)	0.908	0.613
HMDB01886	3-methylxanthine	1 (0.91-1.09)	0.915	0.784	1.02 (0.93-1.12)	0.658	0.609
HMDB12102	C20:0 SM	1 (0.91-1.11)	0.925	0.784	1.02 (0.92-1.13)	0.698	0.609
HMDB13238	C7 carnitine	1 (0.92-1.1)	0.929	0.784	1.02 (0.93-1.11)	0.723	0.609
NA	C20:4 carnitine	1 (0.92-1.1)	0.933	0.784	1.01 (0.92-1.11)	0.762	0.609
NA	C34:2 DAG_or_TAG_fragment	1 (0.91-1.09)	0.935	0.784	1.05 (0.95-1.15)	0.339	0.603
HMDB01991	7-methylxanthine	1 (0.91-1.09)	0.937	0.784	1.02 (0.93-1.12)	0.655	0.609
HMDB00853	acetyl-galactosamine	1 (0.91-1.11)	0.945	0.784	1.04 (0.93-1.15)	0.506	0.609
NA	hydroxyectoine	1 (0.91-1.09)	0.954	0.784	0.99 (0.9-1.09)	0.853	0.613
HMDB00884	ribothymidine	1 (0.91-1.09)	0.955	0.784	0.97 (0.89-1.07)	0.555	0.609
HMDB00123	glycine	1 (0.92-1.09)	0.961	0.784	0.98 (0.89-1.08)	0.649	0.609

HMDB00904	citrulline	1 (0.91-1.09)	0.964	0.784	0.99 (0.9-1.09)	0.864	0.613
HMDB07973*	C34:2 PC	1 (0.91-1.1)	0.965	0.784	1 (0.9-1.1)	0.932	0.613
HMDB00177	histidine	1 (0.9-1.1)	0.968	0.784	1 (0.9-1.11)	0.942	0.613
HMDB02820	methylimidazole acetic acid	1 (0.92-1.09)	0.978	0.789	0.99 (0.91-1.09)	0.879	0.613
NA	C36:3 DAG_or_TAG_fragment	1 (0.92-1.09)	0.994	0.798	1.07 (0.97-1.18)	0.179	0.596

Supplemental Table 2. : Odds ratios (OR) of breast cancer and 95% confidence intervals (CI) per 1 SD increase for all metabolites among premenopausal women at blood collection.

Simple model: adjusts for matching factors including menopause status at blood draw, time of blood draw, date/season of blood draw, luteal day at blood draw, fasting status at blood draw, menopausal status at diagnosis and race.

Adjusted model: in addition to matching factors, this model adjusts for BMI at age 18, weight change between age 18 and time of blood draw, age at menarche, parity and age at first birth, family history of breast cancer, personal history of benign breast disease, physical activity, alcohol consumption, exogenous hormone use, breast feeding history.

**Premenopausal at blood draw: cases = 838, controls = 838**		**Simple Model**		**Adjusted Model**	
**HMDB ID**	**Metabolite**	**OR (95% CI)**	**PVAL**	**OR (95% CI)**	**PVAL**
NA	2-aminohippuric acid	0.82 (0.74-0.9)	<0.001	0.82 (0.73-0.91)	<0.001
HMDB29377	piperine	0.84 (0.76-0.92)	<0.001	0.82 (0.74-0.91)	<0.001
NA	DMGV	0.83 (0.76-0.92)	<0.001	0.86 (0.77-0.96)	0.007
HMDB00715	kynurenic acid	0.84 (0.75-0.93)	0.001	0.82 (0.73-0.92)	0.001
HMDB11394*	C40:7 PE plasmalogen	1.15 (1.04-1.26)	0.007	1.12 (1.01-1.25)	0.03
HMDB00168	asparagine	1.14 (1.03-1.27)	0.014	1.12 (1-1.25)	0.051
HMDB11211*	C34:3 PC plasmalogen	1.13 (1.02-1.25)	0.016	1.11 (0.99-1.24)	0.064
HMDB06344	phenylacetylglutamine	0.88 (0.8-0.98)	0.018	0.9 (0.81-1)	0.055
HMDB01257	spermidine	1.14 (1.02-1.27)	0.022	1.14 (1.02-1.29)	0.023
HMDB10391*	C20:1 LPC	1.14 (1.02-1.27)	0.023	1.12 (1-1.26)	0.052
HMDB04400	5-acetylamino-6-amino-3-methyluracil	0.9 (0.81-0.99)	0.025	0.91 (0.82-1)	0.06
HMDB11103	1,7-dimethyluric acid	0.89 (0.81-0.99)	0.026	0.9 (0.81-1)	0.056
HMDB01855	5-hydroxytryptophol	1.14 (1.01-1.27)	0.03	1.11 (0.99-1.25)	0.08
HMDB04824	N2,N2-dimethylguanosine	0.89 (0.81-0.99)	0.035	0.91 (0.81-1.01)	0.086
HMDB02000	myristoleic acid	0.9 (0.81-0.99)	0.037	0.92 (0.83-1.03)	0.137
HMDB01565	phosphocholine	1.12 (1.01-1.24)	0.037	1.08 (0.97-1.21)	0.17
HMDB32390	2-methyl-4,5-benzoxazole	0.9 (0.82-1)	0.044	0.91 (0.82-1.01)	0.078
HMDB01276	N1-acetylspermidine	1.11 (1-1.23)	0.051	1.11 (1-1.24)	0.061
HMDB13325	C10:2 carnitine	0.91 (0.82-1)	0.054	0.92 (0.83-1.02)	0.12
HMDB00064	creatine	1.1 (0.99-1.22)	0.067	1.13 (1.01-1.26)	0.032
HMDB10404	C22:6 LPC	1.1 (0.99-1.22)	0.07	1.06 (0.95-1.18)	0.336
HMDB10386*	C18:2 LPC	1.1 (0.99-1.22)	0.081	1.06 (0.94-1.18)	0.348
HMDB11503	C16:0 LPE	1.09 (0.99-1.21)	0.087	1.07 (0.96-1.2)	0.208
HMDB11229*	C38:7 PC plasmalogen	1.09 (0.98-1.2)	0.099	1.05 (0.95-1.17)	0.345
HMDB10407*	C16:1 LPC plasmalogen	1.1 (0.98-1.22)	0.1	1.08 (0.96-1.21)	0.204

HMDB13287	N6,N6-dimethyllysine	0.92 (0.83-1.02)	0.109	0.92 (0.83-1.02)	0.097
HMDB11526	C22:6 LPE	1.09 (0.98-1.21)	0.12	1.06 (0.95-1.19)	0.271
HMDB10169	C16:0 SM	1.08 (0.98-1.2)	0.137	1.09 (0.97-1.21)	0.136
HMDB11511	C20:0 LPE	1.08 (0.97-1.2)	0.143	1.08 (0.96-1.22)	0.178
HMDB00767	pseudouridine	0.93 (0.84-1.03)	0.152	0.95 (0.85-1.06)	0.336
HMDB15168	cerulenin	0.93 (0.84-1.03)	0.156	0.93 (0.84-1.04)	0.195
HMDB11745	N-acetylmethionine	1.08 (0.97-1.2)	0.157	1.08 (0.97-1.21)	0.162
HMDB01563	1-methylguanosine	0.93 (0.83-1.03)	0.158	0.94 (0.84-1.05)	0.303
HMDB01859	acetaminophen	0.93 (0.84-1.03)	0.159	0.9 (0.81-1.01)	0.067
HMDB11517	C20:4 LPE	1.08 (0.97-1.19)	0.162	1.08 (0.97-1.2)	0.154
HMDB00562	creatinine	0.93 (0.84-1.03)	0.172	0.92 (0.83-1.02)	0.125
HMDB07883*	C34:4 PC	0.93 (0.84-1.03)	0.172	0.93 (0.84-1.04)	0.203
HMDB00544	5-hydroxymethyl-4-methyluracil	0.93 (0.83-1.03)	0.175	0.95 (0.84-1.06)	0.364
HMDB00991*	2-aminooctanoic acid	1.07 (0.97-1.18)	0.176	1.06 (0.96-1.18)	0.235
HMDB13678	4-hydroxyhippurate	1.07 (0.97-1.19)	0.177	1.07 (0.97-1.19)	0.184
HMDB00883	valine	0.93 (0.85-1.03)	0.178	0.96 (0.86-1.07)	0.501
HMDB06347	C26 carnitine	1.07 (0.97-1.18)	0.184	1.08 (0.98-1.19)	0.133
HMDB00133	guanosine	0.94 (0.85-1.03)	0.185	0.94 (0.85-1.04)	0.218
HMDB01906*	aminoisobutyric acid	1.07 (0.97-1.19)	0.185	1.05 (0.95-1.17)	0.341
HMDB11253*	C38:5 PE plasmalogen	1.07 (0.97-1.18)	0.187	1.08 (0.98-1.2)	0.122
HMDB13127	C4-OH carnitine	1.07 (0.97-1.18)	0.197	1.13 (1.01-1.26)	0.034
HMDB01539	ADMA	0.93 (0.84-1.04)	0.2	0.93 (0.83-1.04)	0.181
NA	C46:2 TAG	0.94 (0.85-1.03)	0.201	0.95 (0.86-1.05)	0.332
HMDB11507*	C18:2 LPE	1.07 (0.97-1.18)	0.203	1.06 (0.95-1.17)	0.312
HMDB00128	guanidinoacetic acid	1.07 (0.97-1.18)	0.205	1.07 (0.96-1.19)	0.244
HMDB11506*	C18:1 LPE	1.07 (0.97-1.18)	0.205	1.05 (0.95-1.17)	0.356
HMDB00853	N-acetylgalactosamine	1.07 (0.96-1.2)	0.206	1.11 (0.99-1.25)	0.066
HMDB11420*	C38:7 PE plasmalogen	1.07 (0.97-1.18)	0.206	1.05 (0.94-1.16)	0.408
NA	C36:2 PS plasmalogen	1.07 (0.96-1.18)	0.213	1.08 (0.97-1.2)	0.169
HMDB15150	sulfamethoxazole	1.09 (0.95-1.25)	0.224	1.09 (0.94-1.25)	0.261
HMDB02802	cortisone	0.94 (0.85-1.04)	0.225	0.94 (0.85-1.05)	0.288
HMDB00687	leucine	0.94 (0.85-1.04)	0.227	0.96 (0.87-1.07)	0.497
HMDB05923	N4-acetylcytidine	0.94 (0.85-1.04)	0.228	0.98 (0.88-1.09)	0.668
HMDB01396	glycocholate	1.06 (0.96-1.18)	0.23	1.07 (0.96-1.19)	0.227

HMDB00054	bilirubin	0.94 (0.86-1.04)	0.232	0.94 (0.85-1.04)	0.226
HMDB00248	thyroxine	0.94 (0.85-1.04)	0.236	0.97 (0.87-1.08)	0.575
HMDB00631*	glycodeoxycholate/glycochenodeoxycholate	1.06 (0.96-1.18)	0.247	1.07 (0.96-1.19)	0.211
HMDB00210	pantothenate	1.06 (0.96-1.18)	0.259	1.06 (0.95-1.18)	0.331
HMDB01414	putrescine	1.06 (0.95-1.18)	0.269	1.04 (0.93-1.17)	0.459
HMDB00172	isoleucine	0.95 (0.86-1.04)	0.271	0.97 (0.88-1.08)	0.623
HMDB10384	C18:0 LPC	1.06 (0.95-1.18)	0.277	1.06 (0.95-1.19)	0.285
NA	C46:3 TAG	0.95 (0.86-1.04)	0.281	0.96 (0.87-1.07)	0.472
HMDB05066	C14 carnitine	1.06 (0.96-1.16)	0.282	1.08 (0.97-1.2)	0.146
HMDB00043	betaine	1.06 (0.95-1.19)	0.288	1.05 (0.93-1.18)	0.404
HMDB11441*	C36:3 PE plasmalogen	1.05 (0.95-1.17)	0.295	1.04 (0.94-1.16)	0.436
HMDB00630	cytosine	0.95 (0.86-1.05)	0.3	0.94 (0.85-1.04)	0.253
HMDB11410*	C36:5 PE plasmalogen	1.05 (0.95-1.16)	0.303	1.06 (0.96-1.17)	0.27
HMDB00206	N6-acetyllysine	0.95 (0.85-1.05)	0.307	0.95 (0.85-1.06)	0.324
HMDB00714	hippurate	1.05 (0.95-1.16)	0.325	1.02 (0.92-1.13)	0.646
HMDB00259	serotonin	1.05 (0.95-1.16)	0.336	1.04 (0.94-1.15)	0.461
HMDB11220*	C36:5 PC plasmalogen	1.05 (0.95-1.16)	0.337	1.04 (0.94-1.15)	0.454
NA	C34:1 DAG_or_TAG_fragment	0.95 (0.86-1.06)	0.346	0.99 (0.89-1.12)	0.913
HMDB02820	methylimidazole acetic acid	0.95 (0.87-1.05)	0.347	0.94 (0.85-1.04)	0.259
HMDB08952*	C34:2 PE plasmalogen	1.05 (0.95-1.16)	0.36	1.03 (0.93-1.14)	0.589
HMDB03357	N-acetylornithine	1.05 (0.95-1.16)	0.362	1.02 (0.92-1.14)	0.658
HMDB00092	dimethylglycine	1.05 (0.95-1.15)	0.372	1.06 (0.96-1.18)	0.248
HMDB01991	7-methylxanthine	0.96 (0.87-1.06)	0.381	0.98 (0.88-1.09)	0.678
HMDB13326	C12:1 carnitine	0.96 (0.87-1.06)	0.393	0.98 (0.88-1.08)	0.674
NA	C44:2 TAG	0.96 (0.87-1.06)	0.396	0.97 (0.88-1.07)	0.534
HMDB34169	methyl N-methylanthranilate	1.04 (0.94-1.15)	0.41	1.04 (0.93-1.15)	0.493
HMDB01431	pyridoxamine	0.95 (0.85-1.07)	0.412	0.98 (0.87-1.11)	0.754
HMDB01847	caffeine	0.96 (0.87-1.06)	0.415	0.97 (0.87-1.07)	0.532
HMDB00301	urocanic acid	0.95 (0.85-1.07)	0.415	0.99 (0.88-1.11)	0.865
NA	C36:2 DAG_or_TAG_fragment	0.96 (0.86-1.06)	0.422	1.01 (0.91-1.13)	0.805
NA	C34:3 DAG_or_TAG_fragment	0.96 (0.87-1.06)	0.436	1.01 (0.91-1.13)	0.797
HMDB04030	21-deoxycortisol	0.96 (0.87-1.06)	0.437	0.95 (0.86-1.06)	0.358
HMDB02815*	C18:1 LPC	1.04 (0.94-1.16)	0.439	1 (0.9-1.12)	0.944
HMDB10395	C20:4 LPC	1.04 (0.94-1.15)	0.44	1.03 (0.92-1.14)	0.651

NA	C34:2 DAG_or_TAG_fragment	0.96 (0.87-1.06)	0.44	1.02 (0.91-1.14)	0.751
HMDB13713	N-acetyltryptophan	0.96 (0.88-1.06)	0.442	0.94 (0.85-1.04)	0.227
HMDB02172	diacetylspermine	1.04 (0.94-1.15)	0.445	1.06 (0.96-1.18)	0.243
HMDB04949	C16:0 Ceramide (d18:1)	1.04 (0.94-1.16)	0.447	1.07 (0.96-1.19)	0.252
HMDB10382	C16:0 LPC	1.04 (0.94-1.16)	0.45	1.04 (0.93-1.16)	0.528
HMDB04620	N-alpha-acetylarginine	0.96 (0.87-1.06)	0.45	0.97 (0.87-1.08)	0.548
HMDB01348	C18:0 SM	1.04 (0.94-1.16)	0.458	1.08 (0.96-1.2)	0.202
HMDB00289	urate	0.97 (0.88-1.06)	0.47	0.98 (0.89-1.08)	0.69
HMDB04620	N-alpha-acetyarginine	0.96 (0.87-1.06)	0.474	0.97 (0.87-1.08)	0.553
HMDB04193	N1-methyl-2-pyridone-5-carboxamide	0.96 (0.87-1.07)	0.474	0.97 (0.87-1.08)	0.581
HMDB00187	serine	1.04 (0.94-1.15)	0.476	1.04 (0.93-1.16)	0.5
HMDB00925	trimethylamine-N-oxide	1.04 (0.94-1.14)	0.483	1.07 (0.97-1.18)	0.194
HMDB01008	biliverdin	1.03 (0.94-1.13)	0.484	1.03 (0.94-1.14)	0.491
HMDB02013	C4 carnitine	0.97 (0.88-1.06)	0.497	0.96 (0.87-1.07)	0.484
NA	threosphingosine	0.96 (0.86-1.07)	0.5	0.99 (0.88-1.11)	0.811
HMDB10383*	C16:1 LPC	0.96 (0.87-1.07)	0.504	0.97 (0.87-1.08)	0.619
HMDB00651	C10 carnitine	0.97 (0.87-1.07)	0.504	0.98 (0.88-1.09)	0.712
HMDB00033	carnosine	0.97 (0.88-1.07)	0.506	0.96 (0.86-1.06)	0.417
HMDB00159	phenylalanine	1.04 (0.93-1.15)	0.507	1.05 (0.95-1.18)	0.338
HMDB02172	N1,N12-diacetylspermine	1.03 (0.94-1.14)	0.515	1.06 (0.95-1.17)	0.302
HMDB00001	1-methylhistidine	0.97 (0.88-1.07)	0.518	0.95 (0.86-1.05)	0.322
HMDB00479	methylhistidine	0.97 (0.88-1.07)	0.521	0.95 (0.86-1.06)	0.352
HMDB00824	C3 carnitine	0.97 (0.88-1.07)	0.525	0.99 (0.89-1.1)	0.857
HMDB00884	ribothymidine	0.97 (0.88-1.07)	0.528	0.95 (0.86-1.06)	0.374
HMDB11756	N-acetylleucine	1.03 (0.93-1.15)	0.536	1.08 (0.97-1.21)	0.175
HMDB01886	3-methylxanthine	0.97 (0.88-1.07)	0.538	1 (0.9-1.11)	0.933
HMDB00791	C8 carnitine	0.97 (0.88-1.07)	0.539	0.99 (0.89-1.1)	0.797
HMDB00812	N-acetylaspartic acid	1.03 (0.93-1.15)	0.548	1.03 (0.92-1.15)	0.572
HMDB00289	uric acid	0.97 (0.89-1.07)	0.555	0.99 (0.9-1.09)	0.811
HMDB59824	4-hydroxy-3-methylacetophenone	0.97 (0.87-1.08)	0.558	0.99 (0.89-1.11)	0.873
HMDB04207	alpha-glutamyllysine	1.04 (0.92-1.16)	0.562	1.06 (0.93-1.19)	0.383
HMDB29416	NMMA	0.97 (0.86-1.09)	0.564	0.99 (0.88-1.13)	0.917
HMDB01325	N6,N6,N6-trimethyllysine	0.97 (0.88-1.08)	0.569	0.97 (0.87-1.08)	0.55
HMDB00705	C6 carnitine	0.97 (0.87-1.08)	0.571	0.99 (0.89-1.11)	0.921

HMDB13331	C14:2 carnitine	0.97 (0.88-1.07)	0.574	0.99 (0.89-1.09)	0.797
HMDB07874*	C32:2 PC	0.97 (0.88-1.07)	0.581	0.98 (0.88-1.09)	0.7
HMDB05015	gabapentin	1.06 (0.86-1.32)	0.582	1.01 (0.81-1.27)	0.918
NA	C16:1 SM	0.97 (0.87-1.08)	0.586	1.02 (0.91-1.15)	0.7
HMDB00696	methionine	1.03 (0.93-1.14)	0.594	1.01 (0.91-1.13)	0.807
HMDB00991*	2-aminooctanoic acid	0.97 (0.86-1.09)	0.604	0.97 (0.86-1.09)	0.597
HMDB00898	1-methylhistamine	1.03 (0.93-1.13)	0.604	1.01 (0.91-1.12)	0.894
HMDB09102*	C38:6 PE	1.03 (0.92-1.15)	0.615	1.02 (0.91-1.14)	0.752
HMDB02064	N-acetylputrescine	0.98 (0.88-1.08)	0.649	0.98 (0.88-1.09)	0.708
HMDB11512*	C20:1 LPE	0.98 (0.88-1.08)	0.651	0.97 (0.87-1.08)	0.584
HMDB00904	citrulline	1.02 (0.92-1.14)	0.652	1.02 (0.92-1.14)	0.668
HMDB03681	4-acetamidobutanoate	0.98 (0.88-1.08)	0.654	0.97 (0.87-1.08)	0.573
HMDB11343*	C34:3 PE plasmalogen	1.02 (0.93-1.13)	0.658	1.01 (0.92-1.12)	0.786
HMDB09012*	C40:6 PE	0.98 (0.88-1.09)	0.659	0.99 (0.88-1.11)	0.827
NA	C20:4 carnitine	1.02 (0.92-1.14)	0.671	1.04 (0.93-1.16)	0.5
HMDB02250	C12 carnitine	0.98 (0.89-1.08)	0.672	1 (0.9-1.11)	0.982
NA	C36:3 DAG_or_TAG_fragment	0.98 (0.89-1.08)	0.673	1.05 (0.94-1.17)	0.417
HMDB00162	proline	1.02 (0.93-1.13)	0.674	1.03 (0.93-1.14)	0.622
HMDB00161	alanine	0.98 (0.88-1.09)	0.682	1 (0.9-1.12)	0.952
NA	hydroxyectoine	1.02 (0.92-1.13)	0.684	1.02 (0.92-1.14)	0.692
HMDB00123	glycine	1.02 (0.92-1.13)	0.687	1.01 (0.91-1.13)	0.846
HMDB10387*	C18:3 LPC	0.98 (0.88-1.09)	0.695	0.97 (0.87-1.09)	0.628
HMDB00688	C5 carnitine	0.98 (0.89-1.08)	0.695	1.01 (0.91-1.13)	0.833
HMDB00063	cortisol	0.98 (0.88-1.09)	0.696	0.97 (0.87-1.08)	0.572
HMDB00897	7-methylguanine	0.98 (0.88-1.09)	0.703	0.97 (0.87-1.08)	0.602
HMDB00630*	cytosine_isomer	1.02 (0.92-1.13)	0.704	1 (0.89-1.11)	0.964
HMDB13130	C5-DC carnitine	0.98 (0.89-1.09)	0.709	0.98 (0.88-1.09)	0.72
NA	C38:5 DAG_or_TAG_fragment	0.98 (0.88-1.09)	0.713	1.03 (0.92-1.15)	0.629
HMDB00719	homoserine	1.02 (0.91-1.15)	0.72	1.03 (0.91-1.17)	0.609
HMDB03334	SDMA	0.98 (0.87-1.1)	0.721	0.96 (0.85-1.08)	0.484
HMDB03282	1-methylguanine	1.02 (0.92-1.13)	0.727	1.03 (0.92-1.14)	0.637
HMDB00679	homocitrulline	1.02 (0.92-1.13)	0.733	1.02 (0.91-1.14)	0.782
HMDB11478*	C18:3 LPE	1.02 (0.92-1.13)	0.745	1 (0.9-1.11)	0.957
HMDB07869*	C30:0 PC	0.98 (0.89-1.09)	0.749	0.96 (0.87-1.07)	0.486

NA	ectoine	0.98 (0.89-1.08)	0.75	0.97 (0.88-1.07)	0.558
HMDB12097	C14:0 SM	0.98 (0.89-1.09)	0.753	0.99 (0.89-1.1)	0.885
HMDB00112	GABA	1.02 (0.92-1.13)	0.754	1.03 (0.92-1.15)	0.586
HMDB00167	threonine	1.02 (0.91-1.14)	0.768	1.03 (0.91-1.16)	0.652
HMDB02014	C14:1 carnitine	0.99 (0.89-1.09)	0.774	1 (0.9-1.11)	0.981
HMDB10403	C22:5 LPC	0.99 (0.89-1.09)	0.779	0.96 (0.86-1.07)	0.449
HMDB00201	C2 carnitine	1.01 (0.92-1.12)	0.782	1.04 (0.93-1.15)	0.485
NA	C36:3 PS plasmalogen	0.99 (0.9-1.08)	0.784	0.99 (0.89-1.09)	0.819
NA	N-methylproline	1.01 (0.92-1.12)	0.786	1 (0.91-1.11)	0.935
HMDB13238	C7 carnitine	1.01 (0.92-1.12)	0.796	1.03 (0.92-1.14)	0.623
HMDB08928*	C34:2 PE	0.99 (0.89-1.09)	0.822	1.01 (0.9-1.12)	0.926
HMDB00716	pipecolic acid	1.01 (0.91-1.12)	0.824	1.02 (0.91-1.14)	0.788
HMDB00182	lysine	0.99 (0.88-1.11)	0.828	1.02 (0.9-1.15)	0.777
Internal Standard	phenylalanine-d8	1.01 (0.9-1.14)	0.829	0.99 (0.88-1.12)	0.889
HMDB04827	proline betaine	0.99 (0.9-1.09)	0.836	0.97 (0.87-1.07)	0.512
HMDB01476	3-hydroxyanthranilic acid	1.01 (0.91-1.12)	0.836	1.02 (0.92-1.14)	0.676
HMDB09003*	C38:4 PE	0.99 (0.9-1.09)	0.842	1.02 (0.92-1.14)	0.678
HMDB00026	N-carbamoyl-beta-alanine	0.99 (0.89-1.1)	0.843	0.97 (0.87-1.08)	0.537
HMDB04953*	C24:1 Ceramide (d18:1)	0.99 (0.89-1.1)	0.848	1 (0.9-1.12)	0.931
HMDB12102	C20:0 SM	1.01 (0.91-1.12)	0.859	1.03 (0.92-1.15)	0.653
HMDB08937*	C36:4 PE	1.01 (0.91-1.12)	0.863	1.02 (0.91-1.14)	0.775
HMDB13631	oleoyl glycine	0.99 (0.9-1.1)	0.865	1 (0.9-1.11)	0.984
HMDB10379	C14:0 LPC	0.99 (0.9-1.09)	0.869	1 (0.9-1.11)	0.97
HMDB00853	acetyl-galactosamine	1.01 (0.9-1.13)	0.871	1.04 (0.92-1.17)	0.523
HMDB00641	glutamine	1.01 (0.91-1.12)	0.872	0.97 (0.87-1.08)	0.588
HMDB00699	1-methylnicotinamide	0.99 (0.9-1.1)	0.886	0.97 (0.87-1.09)	0.635
HMDB08994*	C36:2 PE	0.99 (0.9-1.1)	0.893	1.02 (0.91-1.13)	0.77
NA	C36:4 DAG_or_TAG_fragment	0.99 (0.9-1.09)	0.894	1.05 (0.95-1.17)	0.352
HMDB00177	histidine	1.01 (0.9-1.12)	0.9	1.02 (0.9-1.14)	0.799
HMDB02366	C5:1 carnitine	0.99 (0.9-1.1)	0.904	0.98 (0.88-1.09)	0.726
NA	glutamic acid amide	1.01 (0.91-1.12)	0.913	0.97 (0.87-1.08)	0.551
HMDB13288	C9 carnitine	0.99 (0.9-1.1)	0.918	1 (0.9-1.1)	0.925
HMDB00725	hydroxyproline	1 (0.91-1.11)	0.92	1.01 (0.91-1.12)	0.838
NA	C34:1 DAG_or_TAG_fragment	1 (0.9-1.1)	0.923	1.06 (0.95-1.18)	0.309

Internal Standard	valine-d8	1.01 (0.9-1.13)	0.928	1 (0.89-1.12)	0.965
HMDB00929	tryptophan	1 (0.9-1.1)	0.93	0.99 (0.89-1.1)	0.792
HMDB08925*	C34:0 PE	1 (0.91-1.1)	0.937	0.99 (0.9-1.1)	0.885
HMDB00875	trigonelline	1 (0.91-1.11)	0.948	0.99 (0.89-1.1)	0.861
HMDB07973*	C34:2 PC	1 (0.9-1.11)	0.961	1 (0.89-1.12)	0.994
HMDB00158	tyrosine	1 (0.9-1.11)	0.962	1.01 (0.91-1.13)	0.796
HMDB12103	C22:0 SM	1 (0.89-1.12)	0.966	1.02 (0.9-1.16)	0.735
HMDB00062	carnitine	1 (0.9-1.11)	0.97	1.01 (0.91-1.12)	0.87
HMDB32055	N-acetylhistidine	1 (0.9-1.11)	0.979	0.99 (0.89-1.11)	0.919
HMDB00462	allantoin	1 (0.9-1.12)	0.981	1.04 (0.93-1.17)	0.461
HMDB05862	2-methylguanosine	1 (0.9-1.12)	0.982	1.02 (0.91-1.14)	0.767
NA	C34:2 DAG_or_TAG_fragment	1 (0.91-1.1)	0.989	1.05 (0.94-1.16)	0.382

Supplemental Table 3. : Odds ratios (OR) of breast cancer and 95% confidence intervals (CI) per 1 SD increase for all metabolites, by ER status.

Simple model: adjusts for matching factors including menopause status at blood draw, time of blood draw, date/season of blood draw, luteal day at blood draw, fasting status at blood draw, menopausal status at diagnosis and race.

Adjusted model: in addition to matching factors, this model adjusts for BMI at age 18, weight change between age 18 and time of blood draw, age at menarche, parity and age at first birth, family history of breast cancer, personal history of benign breast disease, physical activity, alcohol consumption, exogenous hormone use, breast feeding history.

Simple Model						
		ER positive		ER negative		
HMDB_ID	METABOLITE	OR (95% CI)	PVAL	OR (95% CI)	PVAL	p.het
HMDB00043	betaine	1.06 (0.95-1.18)	0.309	0.89 (0.73-1.08)	0.241	0.039
HMDB00168	asparagine	1.08 (0.97-1.2)	0.177	1.25 (1.03-1.52)	0.023	0.024
HMDB03681	4-acetamidobutanoate	0.98 (0.88-1.09)	0.701	0.88 (0.72-1.07)	0.214	0.067
HMDB00177	histidine	1.01 (0.91-1.13)	0.793	1.05 (0.86-1.28)	0.62	0.039
HMDB00187	serine	1.01 (0.91-1.12)	0.845	1.13 (0.94-1.36)	0.212	0.023
HMDB00133	guanosine	1.04 (0.93-1.16)	0.487	0.92 (0.79-1.09)	0.302	0.05
HMDB01414	putrescine	1.09 (0.99-1.22)	0.092	1 (0.84-1.21)	0.999	0.061
HMDB00167	threonine	1 (0.89-1.13)	0.966	1.11 (0.9-1.38)	0.325	0.111
HMDB13325	C10:2 carnitine	0.96 (0.87-1.07)	0.501	0.88 (0.74-1.06)	0.174	0.173
Internal Standard	phenylalanine-d8	1.03 (0.94-1.15)	0.533	1.08 (0.9-1.35)	0.455	0.153
HMDB00719	homoserine	1 (0.89-1.13)	0.965	1.13 (0.91-1.4)	0.282	0.113
HMDB00875	trigonelline	0.95 (0.86-1.06)	0.382	0.92 (0.76-1.11)	0.375	0.185
HMDB02172	N1,N12-diacetylspermine	0.99 (0.89-1.1)	0.836	1.22 (1.02-1.46)	0.029	0.074
HMDB15168	cerulenin	0.99 (0.9-1.1)	0.899	0.95 (0.8-1.14)	0.567	0.187
HMDB00898	1-methylhistamine	1.08 (0.97-1.19)	0.144	0.92 (0.77-1.11)	0.39	0.197
HMDB03282	1-methylguanine	1.02 (0.92-1.13)	0.687	0.9 (0.76-1.07)	0.235	0.179
HMDB00210	pantothenate	1.06 (0.95-1.17)	0.279	1.14 (0.94-1.37)	0.171	0.159
HMDB12103	C22:0 SM	1.01 (0.91-1.12)	0.905	0.98 (0.81-1.18)	0.797	0.164
NA	2-aminohippuric acid	0.9 (0.81-0.99)	0.041	0.79 (0.65-0.96)	0.016	0.141
HMDB11441*	C36:3 PE plasmalogen	1.03 (0.93-1.14)	0.542	1.09 (0.91-1.32)	0.358	0.149
HMDB13288	C9 carnitine	1.05 (0.94-1.16)	0.376	0.89 (0.73-1.07)	0.207	0.155
HMDB04949	C16:0 Ceramide (d18:1)	1.01 (0.91-1.12)	0.852	1.03 (0.87-1.26)	0.787	0.147
HMDB11343*	C34:3 PE plasmalogen	1.01 (0.92-1.12)	0.787	1.01 (0.84-1.22)	0.92	0.199
HMDB01476	3-hydroxyanthranilic acid	0.97 (0.88-1.08)	0.616	1.05 (0.88-1.28)	0.579	0.183
HMDB00182	lysine	0.98 (0.87-1.1)	0.731	0.98 (0.79-1.2)	0.823	0.137
HMDB11745	N-acetylmethionine	1.06 (0.96-1.18)	0.242	1.12 (0.93-1.35)	0.25	0.164
HMDB01348	C18:0 SM	1.03 (0.93-1.15)	0.555	0.96 (0.8-1.15)	0.654	0.27
HMDB11211*	C34:3 PC plasmalogen	1.09 (0.98-1.21)	0.097	1.06 (0.88-1.28)	0.562	0.189
HMDB00897	7-methylguanine	0.98 (0.89-1.09)	0.723	0.9 (0.75-1.09)	0.288	0.366
HMDB00696	methionine	1 (0.9-1.11)	0.985	1.15 (0.95-1.38)	0.145	0.148
HMDB00925	trimethylamine-N-oxide	1.01 (0.91-1.11)	0.918	0.94 (0.78-1.14)	0.556	0.212
HMDB11253*	C38:5 PE plasmalogen	0.99 (0.9-1.1)	0.888	0.98 (0.82-1.18)	0.828	0.364
HMDB00159	phenylalanine	0.99 (0.89-1.1)	0.86	1.08 (0.89-1.29)	0.434	0.172
HMDB00064	creatine	1.06 (0.95-1.19)	0.271	1.21 (0.99-1.49)	0.06	0.212
HMDB12102	C20:0 SM	1.01 (0.91-1.12)	0.848	0.99 (0.81-1.2)	0.899	0.241
HMDB11410*	C36:5 PE plasmalogen	1.01 (0.91-1.12)	0.863	0.97 (0.8-1.17)	0.729	0.382
HMDB00248	thyroxine	0.98 (0.88-1.09)	0.7	1.04 (0.86-1.29)	0.672	0.154
HMDB13326	C12:1 carnitine	1.02 (0.92-1.14)	0.726	0.87 (0.72-1.06)	0.161	0.253
HMDB32390	2-methyl-4,5-benzoxazole	0.9 (0.81-0.99)	0.035	1 (0.84-1.22)	0.968	0.168
HMDB01855	5-hydroxytryptophol	1.13 (1.02-1.26)	0.018	0.99 (0.82-1.21)	0.919	0.294
HMDB00904	citrulline	0.98 (0.88-1.09)	0.712	1.08 (0.89-1.31)	0.436	0.388
HMDB01539	ADMA	0.96 (0.86-1.06)	0.418	0.96 (0.8-1.16)	0.685	0.242
NA	C36:2 DAG_or_TAG_fragment	0.96 (0.86-1.06)	0.419	0.93 (0.77-1.13)	0.475	0.36
HMDB02064	N-acetylputrescine	1 (0.9-1.11)	0.939	0.98 (0.81-1.19)	0.818	0.313
HMDB00161	alanine	0.97 (0.87-1.08)	0.591	1.05 (0.86-1.28)	0.658	0.22
HMDB13130	C5-DC carnitine	0.97 (0.87-1.07)	0.529	1.04 (0.86-1.26)	0.682	0.458
HMDB05015	gabapentin	1.05 (0.95-1.16)	0.388	0.93 (0.76-1.14)	0.512	0.239
HMDB09003*	C38:4 PE	0.94 (0.84-1.05)	0.289	0.93 (0.76-1.14)	0.492	0.378
HMDB00001	1-methylhistidine	0.94 (0.85-1.05)	0.278	1.1 (0.91-1.33)	0.299	0.384
NA	hydroxyectoine	0.99 (0.89-1.1)	0.846	1.06 (0.88-1.28)	0.552	0.459
HMDB00112	GABA	1.02 (0.91-1.13)	0.75	1.11 (0.92-1.35)	0.286	0.206

Adjusted Model						
		ER positive		ER negative		
HMDB_ID	METABOLITE	OR (95% CI)	PVAL	OR (95% CI)	PVAL	p.het
HMDB00043	betaine	1.07 (0.96-1.2)	0.22	0.89 (0.73-1.09)	0.253	0.016
HMDB00168	asparagine	1.06 (0.94-1.18)	0.346	1.21 (0.99-1.47)	0.063	0.027
HMDB03681	4-acetamidobutanoate	0.97 (0.87-1.08)	0.558	0.89 (0.72-1.09)	0.257	0.031
HMDB00177	histidine	1.01 (0.91-1.13)	0.858	1.06 (0.87-1.29)	0.591	0.047
HMDB00187	serine	1.02 (0.92-1.13)	0.712	1.1 (0.91-1.33)	0.337	0.059
HMDB00133	guanosine	1.04 (0.94-1.16)	0.464	0.94 (0.8-1.11)	0.439	0.061
HMDB01414	putrescine	1.08 (0.97-1.2)	0.175	1.01 (0.85-1.23)	0.91	0.094
HMDB00167	threonine	1 (0.88-1.12)	0.938	1.12 (0.9-1.39)	0.312	0.097
HMDB13325	C10:2 carnitine	0.97 (0.87-1.08)	0.591	0.9 (0.76-1.08)	0.254	0.097
Internal Standard	phenylalanine-d8	1.03 (0.94-1.15)	0.511	1.09 (0.91-1.36)	0.407	0.099
HMDB00719	homoserine	1 (0.88-1.12)	0.947	1.13 (0.91-1.41)	0.265	0.099
HMDB00875	trigonelline	0.94 (0.84-1.05)	0.259	0.89 (0.73-1.08)	0.222	0.102
HMDB02172	N1,N12-diacetylspermine	1 (0.9-1.12)	0.961	1.27 (1.05-1.52)	0.01	0.111
HMDB15168	cerulenin	0.99 (0.89-1.1)	0.822	0.96 (0.8-1.16)	0.676	0.119
HMDB00898	1-methylhistamine	1.07 (0.96-1.18)	0.222	0.93 (0.77-1.12)	0.44	0.12
HMDB03282	1-methylguanine	1.03 (0.93-1.14)	0.598	0.92 (0.77-1.1)	0.354	0.12
HMDB00210	pantothenate	1.05 (0.94-1.17)	0.386	1.16 (0.96-1.41)	0.117	0.123
HMDB12103	C22:0 SM	1.02 (0.92-1.14)	0.719	1 (0.83-1.22)	0.979	0.126
NA	2-aminohippuric acid	0.9 (0.81-1)	0.05	0.82 (0.67-1)	0.046	0.127
HMDB11441*	C36:3 PE plasmalogen	1.03 (0.92-1.14)	0.629	1.07 (0.88-1.3)	0.52	0.13
HMDB13288	C9 carnitine	1.03 (0.93-1.15)	0.531	0.88 (0.73-1.07)	0.204	0.132
HMDB04949	C16:0 Ceramide (d18:1)	1.01 (0.91-1.12)	0.896	1.06 (0.89-1.31)	0.584	0.144
HMDB11343*	C34:3 PE plasmalogen	1 (0.9-1.11)	0.948	0.99 (0.82-1.2)	0.954	0.148
HMDB01476	3-hydroxyanthranilic acid	0.96 (0.87-1.06)	0.428	1.05 (0.88-1.28)	0.581	0.149
HMDB00182	lysine	0.99 (0.88-1.11)	0.838	1.02 (0.83-1.27)	0.841	0.156
HMDB11745	N-acetylmethionine	1.06 (0.96-1.18)	0.251	1.11 (0.92-1.34)	0.276	0.167
HMDB01348	C18:0 SM	1.05 (0.94-1.17)	0.407	1 (0.83-1.21)	0.978	0.167
HMDB11211*	C34:3 PC plasmalogen	1.07 (0.96-1.2)	0.232	0.99 (0.81-1.21)	0.909	0.167
HMDB00897	7-methylguanine	0.97 (0.88-1.08)	0.609	0.91 (0.75-1.09)	0.293	0.192
HMDB00696	methionine	0.99 (0.89-1.1)	0.801	1.15 (0.95-1.38)	0.141	0.193
HMDB00925	trimethylamine-N-oxide	1.02 (0.92-1.13)	0.715	0.95 (0.79-1.15)	0.626	0.193
HMDB11253*	C38:5 PE plasmalogen	1.01 (0.91-1.12)	0.837	1 (0.83-1.2)	0.959	0.201
HMDB00159	phenylalanine	1 (0.9-1.11)	0.996	1.13 (0.93-1.37)	0.208	0.209
HMDB00064	creatine	1.07 (0.96-1.2)	0.208	1.26 (1.03-1.55)	0.03	0.21
HMDB12102	C20:0 SM	1.01 (0.91-1.13)	0.856	1.01 (0.83-1.24)	0.896	0.214
HMDB11410*	C36:5 PE plasmalogen	1.01 (0.91-1.13)	0.783	0.98 (0.81-1.19)	0.866	0.232
HMDB00248	thyroxine	1 (0.89-1.11)	0.953	1.08 (0.88-1.34)	0.473	0.238
HMDB13326	C12:1 carnitine	1.03 (0.92-1.15)	0.632	0.89 (0.73-1.08)	0.229	0.252
HMDB32390	2-methyl-4,5-benzoxazole	0.9 (0.81-1)	0.044	1.01 (0.84-1.23)	0.921	0.255
HMDB01855	5-hydroxytryptophol	1.11 (1-1.24)	0.059	0.97 (0.8-1.18)	0.741	0.26
HMDB00904	citrulline	0.98 (0.88-1.09)	0.661	1.06 (0.88-1.29)	0.538	0.273
HMDB01539	ADMA	0.96 (0.87-1.07)	0.455	0.98 (0.81-1.19)	0.864	0.282
NA	C36:2 DAG_or_TAG_fragment	0.98 (0.88-1.1)	0.766	1 (0.82-1.22)	0.997	0.295
HMDB02064	N-acetylputrescine	0.98 (0.88-1.1)	0.778	0.99 (0.81-1.21)	0.946	0.3
HMDB00161	alanine	0.99 (0.88-1.11)	0.81	1.1 (0.9-1.36)	0.348	0.306
HMDB13130	C5-DC carnitine	0.96 (0.86-1.07)	0.466	1.05 (0.86-1.27)	0.653	0.309
HMDB05015	gabapentin	1.04 (0.94-1.16)	0.421	0.94 (0.76-1.15)	0.547	0.309
HMDB09003*	C38:4 PE	0.96 (0.85-1.07)	0.456	0.98 (0.79-1.21)	0.835	0.311
HMDB00001	1-methylhistidine	0.93 (0.84-1.04)	0.195	1.11 (0.91-1.33)	0.301	0.313
NA	hydroxyectoine	0.99 (0.89-1.1)	0.801	1.04 (0.86-1.27)	0.671	0.322
HMDB00112	GABA	1.03 (0.92-1.15)	0.581	1.15 (0.95-1.41)	0.16	0.322

HMDB08952*	C34:2 PE plasmalogen	1.03 (0.93-1.14)	0.53	1.01 (0.84-1.21)	0.949	0.436	HMDB08952*	C34:2 PE plasmalogen	1.02 (0.92-1.13)	0.703	0.98 (0.82-1.19)	0.856	0.334
HMDB02000	myristoleic acid	0.95 (0.86-1.05)	0.306	1.01 (0.84-1.23)	0.892	0.59	HMDB02000	myristoleic acid	0.97 (0.88-1.08)	0.601	1.04 (0.86-1.25)	0.71	0.337
HMDB01276	N1-acetylspermidine	1.12 (1-1.24)	0.041	1.11 (0.91-1.34)	0.295	0.706	HMDB01276	N1-acetylspermidine	1.13 (1.01-1.26)	0.029	1.13 (0.93-1.36)	0.217	0.349
NA	C36:3 DAG_or_TAG_fragment	0.99 (0.89-1.1)	0.865	0.96 (0.8-1.16)	0.702	0.572	NA	C36:3 DAG_or_TAG_fragment	1.03 (0.92-1.16)	0.555	1.04 (0.85-1.27)	0.733	0.352
HMDB00462	allantoin	0.94 (0.84-1.04)	0.243	0.94 (0.77-1.14)	0.523	0.414	HMDB00462	allantoin	0.96 (0.86-1.07)	0.454	0.97 (0.79-1.19)	0.765	0.355
HMDB00631*	glycodeoxycholate/glycochenode	0.99 (0.88-1.1)	0.813	0.99 (0.81-1.21)	0.907	0.267	HMDB00631*	glycodeoxycholate/glycocheno	1 (0.9-1.13)	0.935	1 (0.82-1.23)	0.966	0.356
HMDB11220*	C36:5 PC plasmalogen	1.01 (0.91-1.12)	0.87	0.87 (0.72-1.05)	0.152	0.484	HMDB11220*	C36:5 PC plasmalogen	1.01 (0.91-1.12)	0.897	0.86 (0.71-1.04)	0.127	0.357
HMDB12097	C14:0 SM	0.96 (0.87-1.07)	0.507	0.89 (0.73-1.07)	0.212	0.391	HMDB12097	C14:0 SM	0.95 (0.85-1.06)	0.354	0.91 (0.75-1.11)	0.358	0.361
HMDB05862	2-methylguanosine	1.02 (0.92-1.13)	0.707	1.09 (0.92-1.35)	0.402	0.58	HMDB05862	2-methylguanosine	1.02 (0.92-1.14)	0.647	1.12 (0.94-1.4)	0.271	0.365
HMDB13287	N6,N6-dimethyllysine	0.95 (0.85-1.05)	0.294	0.92 (0.76-1.12)	0.41	0.543	HMDB13287	N6,N6-dimethyllysine	0.95 (0.85-1.05)	0.315	0.93 (0.77-1.13)	0.476	0.366
HMDB11512*	C20:1 LPE	0.96 (0.86-1.06)	0.41	1.09 (0.9-1.32)	0.381	0.39	HMDB11512*	C20:1 LPE	0.94 (0.84-1.04)	0.214	1.06 (0.88-1.29)	0.551	0.37
HMDB02172	diacetylspermine	1 (0.9-1.11)	0.978	1.13 (0.94-1.36)	0.205	0.258	HMDB02172	diacetylspermine	1.01 (0.91-1.12)	0.856	1.17 (0.97-1.42)	0.105	0.372
HMDB00123	glycine	1.01 (0.91-1.12)	0.824	1.12 (0.93-1.35)	0.243	0.295	HMDB00123	glycine	1.01 (0.91-1.12)	0.884	1.06 (0.87-1.3)	0.541	0.372
HMDB09102*	C38:6 PE	0.99 (0.88-1.11)	0.854	0.98 (0.8-1.2)	0.859	0.47	HMDB09102*	C38:6 PE	0.96 (0.86-1.08)	0.541	0.98 (0.79-1.2)	0.828	0.374
HMDB04620	N-alpha-acetyarginine	0.96 (0.86-1.07)	0.496	0.99 (0.82-1.21)	0.947	0.313	HMDB04620	N-alpha-acetyarginine	0.97 (0.87-1.08)	0.563	1 (0.83-1.22)	0.985	0.385
HMDB01431	pyridoxamine	0.95 (0.85-1.07)	0.402	0.94 (0.77-1.15)	0.552	0.31	HMDB01431	pyridoxamine	0.96 (0.85-1.08)	0.485	0.98 (0.8-1.22)	0.881	0.396
HMDB15150	sulfamethoxazole	1.02 (0.93-1.13)	0.655	1.12 (0.93-1.36)	0.24	0.686	HMDB15150	sulfamethoxazole	1.02 (0.92-1.14)	0.65	1.14 (0.94-1.39)	0.204	0.396
HMDB13678	4-hydroxyhippurate	1.02 (0.92-1.13)	0.7	1.02 (0.84-1.23)	0.878	0.281	HMDB13678	4-hydroxyhippurate	1.02 (0.92-1.13)	0.737	1.01 (0.83-1.23)	0.893	0.401
HMDB13631	oleoyl glycine	1.02 (0.91-1.13)	0.778	1.04 (0.86-1.27)	0.709	0.527	HMDB13631	oleoyl glycine	1.01 (0.91-1.13)	0.837	1.03 (0.85-1.26)	0.802	0.407
HMDB00715	kynurenic acid	0.91 (0.82-1.01)	0.066	0.83 (0.69-1)	0.056	0.43	HMDB00715	kynurenic acid	0.9 (0.81-1)	0.049	0.83 (0.69-1.01)	0.067	0.409
HMDB11756	N-acetylleucine	0.98 (0.89-1.09)	0.768	1.02 (0.84-1.23)	0.863	0.225	HMDB11756	N-acetylleucine	1 (0.9-1.11)	0.996	1.06 (0.87-1.28)	0.584	0.414
NA	C36:3 PS plasmalogen	0.97 (0.87-1.07)	0.516	1.07 (0.88-1.32)	0.504	0.663	NA	C36:3 PS plasmalogen	0.96 (0.86-1.07)	0.434	1.11 (0.91-1.39)	0.321	0.416
HMDB01008	biliverdin	1.06 (0.96-1.19)	0.303	1.07 (0.88-1.37)	0.559	0.275	HMDB01008	biliverdin	1.06 (0.96-1.19)	0.281	1.05 (0.88-1.33)	0.663	0.421
HMDB00929	tryptophan	1.03 (0.93-1.15)	0.518	0.99 (0.83-1.21)	0.953	0.422	HMDB00929	tryptophan	1.03 (0.93-1.14)	0.618	0.98 (0.82-1.2)	0.87	0.422
HMDB04620	N-alpha-acetylarginine	0.95 (0.85-1.06)	0.357	0.97 (0.8-1.18)	0.768	0.369	HMDB04620	N-alpha-acetylarginine	0.96 (0.86-1.07)	0.436	0.97 (0.8-1.19)	0.783	0.423
HMDB02820	methylimidazole acetic acid	0.96 (0.87-1.06)	0.428	1.07 (0.89-1.3)	0.468	0.611	HMDB02820	methylimidazole acetic acid	0.95 (0.86-1.05)	0.345	1.07 (0.89-1.3)	0.491	0.424
NA	N-methylproline	0.96 (0.86-1.06)	0.396	1.03 (0.85-1.24)	0.778	0.277	NA	N-methylproline	0.96 (0.86-1.07)	0.444	1.03 (0.85-1.24)	0.776	0.428
HMDB03357	N-acetylornithine	0.98 (0.89-1.09)	0.724	1 (0.83-1.21)	0.981	0.185	HMDB03357	N-acetylornithine	0.95 (0.86-1.06)	0.358	0.98 (0.81-1.19)	0.807	0.428
HMDB32055	N-acetylhistidine	0.97 (0.88-1.08)	0.622	1.05 (0.87-1.27)	0.635	0.511	HMDB32055	N-acetylhistidine	0.97 (0.87-1.07)	0.524	1.03 (0.85-1.25)	0.764	0.432
HMDB09012*	C40:6 PE	0.94 (0.84-1.05)	0.273	0.94 (0.77-1.15)	0.571	0.499	HMDB09012*	C40:6 PE	0.93 (0.83-1.05)	0.237	0.98 (0.79-1.21)	0.841	0.433
HMDB02250	C12 carnitine	1.02 (0.91-1.13)	0.78	0.9 (0.74-1.09)	0.273	0.477	HMDB02250	C12 carnitine	1.02 (0.91-1.13)	0.772	0.91 (0.75-1.1)	0.315	0.438
HMDB11511	C20:0 LPE	1.05 (0.94-1.16)	0.402	1.14 (0.94-1.39)	0.176	0.276	HMDB11511	C20:0 LPE	1.04 (0.93-1.16)	0.506	1.11 (0.91-1.36)	0.324	0.44
HMDB00812	N-acetylaspartic acid	1.03 (0.93-1.14)	0.612	0.97 (0.79-1.17)	0.745	0.521	HMDB00812	N-acetylaspartic acid	1.01 (0.91-1.12)	0.843	0.93 (0.76-1.14)	0.495	0.442
HMDB04953*	C24:1 Ceramide (d18:1)	0.99 (0.9-1.1)	0.907	1 (0.84-1.21)	0.983	0.357	HMDB04953*	C24:1 Ceramide (d18:1)	0.98 (0.89-1.09)	0.743	1.01 (0.85-1.22)	0.898	0.449
HMDB03334	SDMA	1.03 (0.93-1.14)	0.556	0.98 (0.81-1.18)	0.801	0.528	HMDB03334	SDMA	1.01 (0.91-1.12)	0.808	0.97 (0.8-1.17)	0.718	0.451
NA	ectoine	1 (0.9-1.11)	0.953	1.1 (0.91-1.33)	0.345	0.39	NA	ectoine	0.99 (0.89-1.1)	0.847	1.1 (0.91-1.35)	0.319	0.453
HMDB00206	N6-acetyllysine	0.96 (0.87-1.06)	0.424	0.95 (0.78-1.15)	0.587	0.478	HMDB00206	N6-acetyllysine	0.96 (0.86-1.06)	0.399	0.96 (0.79-1.16)	0.65	0.456
NA	C16:1 SM	0.99 (0.89-1.1)	0.82	0.86 (0.72-1.04)	0.109	0.534	NA	C16:1 SM	1.01 (0.9-1.13)	0.928	0.91 (0.75-1.1)	0.325	0.459
HMDB00128	guanidinoacetic acid	1.07 (0.96-1.18)	0.212	1 (0.83-1.21)	0.976	0.469	HMDB00128	guanidinoacetic acid	1.07 (0.96-1.19)	0.232	0.95 (0.79-1.15)	0.607	0.461
HMDB00544	5-hydroxymethyl-4-methyluracil	0.92 (0.83-1.02)	0.116	1.06 (0.87-1.28)	0.574	0.703	HMDB00544	5-hydroxymethyl-4-methylurac	0.92 (0.83-1.03)	0.142	1.09 (0.9-1.33)	0.382	0.475
NA	C34:1 DAG_or_TAG_fragment	0.97 (0.88-1.08)	0.602	1 (0.83-1.2)	0.975	0.902	NA	C34:1 DAG_or_TAG_fragment	1.01 (0.9-1.13)	0.864	1.06 (0.87-1.3)	0.537	0.479
HMDB00824	C3 carnitine	0.92 (0.83-1.01)	0.091	1.04 (0.86-1.25)	0.711	0.365	HMDB00824	C3 carnitine	0.93 (0.83-1.03)	0.153	1.09 (0.9-1.32)	0.393	0.488
HMDB02014	C14:1 carnitine	1.03 (0.92-1.14)	0.63	0.93 (0.77-1.13)	0.452	0.512	HMDB02014	C14:1 carnitine	1.03 (0.93-1.15)	0.564	0.94 (0.78-1.14)	0.543	0.506
HMDB02366	C5:1 carnitine	0.99 (0.9-1.1)	0.92	1.01 (0.84-1.22)	0.904	0.594	HMDB02366	C5:1 carnitine	0.99 (0.89-1.09)	0.778	1.02 (0.85-1.24)	0.803	0.508
HMDB00479	methylhistidine	0.95 (0.85-1.05)	0.298	1.09 (0.9-1.31)	0.392	0.606	HMDB00479	methylhistidine	0.94 (0.84-1.04)	0.225	1.09 (0.9-1.32)	0.378	0.509
NA	C34:2 DAG_or_TAG_fragment	0.98 (0.89-1.09)	0.723	0.98 (0.82-1.19)	0.864	0.694	NA	C34:2 DAG_or_TAG_fragment	1.01 (0.9-1.13)	0.885	1.05 (0.86-1.28)	0.653	0.512
HMDB01859	acetaminophen	0.93 (0.84-1.03)	0.169	0.95 (0.77-1.14)	0.578	0.756	HMDB01859	acetaminophen	0.91 (0.82-1.01)	0.081	0.93 (0.76-1.13)	0.496	0.515
HMDB00699	1-methylnicotinamide	1.05 (0.94-1.17)	0.386	1.03 (0.84-1.26)	0.775	0.493	HMDB00699	1-methylnicotinamide	1.01 (0.91-1.13)	0.81	1.01 (0.82-1.25)	0.899	0.515
HMDB13331	C14:2 carnitine	1.01 (0.91-1.13)	0.802	0.91 (0.75-1.1)	0.33	0.635	HMDB13331	C14:2 carnitine	1.02 (0.92-1.14)	0.694	0.92 (0.76-1.11)	0.374	0.518
HMDB08994*	C36:2 PE	0.93 (0.83-1.03)	0.176	0.96 (0.79-1.17)	0.699	0.657	HMDB08994*	C36:2 PE	0.93 (0.83-1.04)	0.221	1 (0.82-1.22)	0.989	0.522
HMDB10407*	C16:1 LPC plasmalogen	1.05 (0.95-1.17)	0.341	1.1 (0.91-1.32)	0.345	0.281	HMDB10407*	C16:1 LPC plasmalogen	1.04 (0.94-1.16)	0.442	1.07 (0.88-1.3)	0.501	0.523
HMDB29416	NMMA	0.99 (0.89-1.11)	0.878	0.92 (0.75-1.13)	0.427	0.602	HMDB29416	NMMA	1 (0.89-1.12)	0.999	0.96 (0.78-1.18)	0.67	0.524
HMDB00853	N-acetylgalactosamine	1.05 (0.95-1.16)	0.343	1.07 (0.89-1.29)	0.462	0.486	HMDB00853	N-acetylgalactosamine	1.08 (0.97-1.2)	0.166	1.1 (0.91-1.34)	0.319	0.533
NA	glutamic acid amide	1.07 (0.97-1.2)	0.19	0.92 (0.76-1.13)	0.421	0.415	NA	glutamic acid amide	1.06 (0.95-1.18)	0.286	0.9 (0.74-1.09)	0.279	0.539
HMDB00259	serotonin	1.01 (0.91-1.12)	0.83	1.03 (0.86-1.25)	0.742	0.953	HMDB00259	serotonin	1.01 (0.91-1.11)	0.922	1.01 (0.84-1.23)	0.904	0.54
HMDB00641	glutamine	1.08 (0.97-1.2)	0.182	0.93 (0.76-1.13)	0.442	0.416	HMDB00641	glutamine	1.06 (0.95-1.19)	0.278	0.9 (0.74-1.1)	0.29	0.543
NA	DMGV	0.86 (0.77-0.95)	0.003	0.83 (0.69-1)	0.043	0.487	NA	DMGV	0.86 (0.76-0.97)	0.011	0.89 (0.72-1.1)	0.261	0.547
HMDB04193	N1-methyl-2-pyridone-5-carboxai	0.97 (0.87-1.07)	0.533	1.05 (0.87-1.27)	0.586	0.411	HMDB04193	N1-methyl-2-pyridone-5-carbo	0.96 (0.86-1.07)	0.431	1.08 (0.89-1.31)	0.43	0.558
HMDB06347	C26 carnitine	1.06 (0.96-1.18)	0.256	0.97 (0.81-1.16)	0.702	0.771	HMDB06347	C26 carnitine	1.07 (0.96-1.19)	0.202	0.97 (0.81-1.17)	0.772	0.558

HMDB08937*	C36:4 PE	0.93 (0.83-1.04)	0.22	0.96 (0.78-1.18)	0.677	0.699	HMDB08937*	C36:4 PE	0.92 (0.82-1.04)	0.17	0.97 (0.79-1.2)	0.788	0.563
NA	C36:2 PS plasmalogen	1.06 (0.95-1.17)	0.288	1.05 (0.87-1.26)	0.627	0.515	NA	C36:2 PS plasmalogen	1.06 (0.96-1.18)	0.265	1.07 (0.89-1.29)	0.482	0.575
HMDB00687	leucine	0.94 (0.85-1.04)	0.262	0.95 (0.79-1.15)	0.604	0.511	HMDB00687	leucine	0.96 (0.86-1.07)	0.491	1.01 (0.83-1.24)	0.902	0.578
NA	C38:5 DAG_or_TAG_fragment	0.95 (0.86-1.06)	0.404	0.9 (0.74-1.1)	0.322	0.631	NA	C38:5 DAG_or_TAG_fragment	0.97 (0.87-1.09)	0.662	0.96 (0.78-1.19)	0.738	0.579
HMDB34169	methyl N-methylanthranilate	1.03 (0.93-1.14)	0.601	1.02 (0.85-1.24)	0.795	0.525	HMDB34169	methyl N-methylanthranilate	1.02 (0.92-1.14)	0.674	1.01 (0.84-1.22)	0.877	0.581
NA	C36:4 DAG_or_TAG_fragment	1 (0.9-1.1)	0.93	1 (0.83-1.21)	0.992	0.95	NA	C36:4 DAG_or_TAG_fragment	1.04 (0.93-1.16)	0.539	1.06 (0.87-1.3)	0.54	0.581
HMDB00853	acetyl-galactosamine	1.01 (0.91-1.12)	0.834	1.08 (0.9-1.31)	0.399	0.673	HMDB00853	acetyl-galactosamine	1.02 (0.92-1.14)	0.657	1.11 (0.91-1.34)	0.297	0.587
HMDB00201	C2 carnitine	0.95 (0.85-1.05)	0.335	1.1 (0.91-1.34)	0.311	0.636	HMDB00201	C2 carnitine	0.96 (0.86-1.07)	0.477	1.14 (0.94-1.38)	0.176	0.587
HMDB00651	C10 carnitine	1.01 (0.9-1.12)	0.878	0.88 (0.73-1.08)	0.219	0.483	HMDB00651	C10 carnitine	1.01 (0.9-1.12)	0.928	0.89 (0.73-1.08)	0.244	0.589
HMDB07874*	C32:2 PC	0.96 (0.86-1.07)	0.447	1.01 (0.84-1.22)	0.93	0.742	HMDB07874*	C32:2 PC	0.95 (0.85-1.06)	0.318	1.03 (0.85-1.26)	0.759	0.613
HMDB11420*	C38:7 PE plasmalogen	1.05 (0.94-1.17)	0.402	0.95 (0.79-1.16)	0.642	0.75	HMDB11420*	C38:7 PE plasmalogen	1.01 (0.9-1.13)	0.861	0.94 (0.77-1.15)	0.553	0.613
HMDB00562	creatinine	0.95 (0.86-1.05)	0.314	0.94 (0.79-1.14)	0.528	0.776	HMDB00562	creatinine	0.94 (0.85-1.05)	0.289	0.93 (0.78-1.12)	0.428	0.62
HMDB00630*	cytosine_isomer	0.97 (0.88-1.08)	0.632	0.97 (0.8-1.18)	0.785	0.796	HMDB00630*	cytosine_isomer	0.95 (0.86-1.06)	0.402	0.95 (0.78-1.16)	0.63	0.621
NA	C34:3 DAG_or_TAG_fragment	0.94 (0.84-1.04)	0.244	0.98 (0.8-1.18)	0.799	0.672	NA	C34:3 DAG_or_TAG_fragment	0.96 (0.85-1.08)	0.497	1.06 (0.86-1.3)	0.609	0.621
HMDB01565	phosphocholine	1.13 (1.02-1.26)	0.022	1.07 (0.88-1.3)	0.525	0.434	HMDB01565	phosphocholine	1.14 (1.02-1.27)	0.026	1.01 (0.82-1.23)	0.956	0.626
HMDB10379	C14:0 LPC	0.95 (0.86-1.06)	0.361	1.05 (0.87-1.27)	0.634	0.537	HMDB10379	C14:0 LPC	0.94 (0.84-1.04)	0.233	1.07 (0.88-1.3)	0.495	0.634
HMDB02802	cortisone	0.95 (0.86-1.06)	0.367	1.04 (0.87-1.29)	0.72	0.555	HMDB02802	cortisone	0.95 (0.86-1.06)	0.368	1.03 (0.86-1.28)	0.789	0.635
HMDB10387*	C18:3 LPC	0.97 (0.87-1.07)	0.5	1.11 (0.92-1.34)	0.285	0.329	HMDB10387*	C18:3 LPC	0.95 (0.86-1.06)	0.358	1.1 (0.91-1.33)	0.327	0.639
HMDB00054	bilirubin	0.98 (0.88-1.09)	0.7	0.98 (0.82-1.21)	0.821	0.777	HMDB00054	bilirubin	0.98 (0.88-1.09)	0.703	0.98 (0.83-1.22)	0.858	0.641
HMDB29377	piperine	0.82 (0.74-0.91)	0	0.84 (0.7-1.02)	0.076	0.797	HMDB29377	piperine	0.79 (0.71-0.88)	0	0.84 (0.69-1.02)	0.076	0.655
NA	threosphingosine	0.96 (0.87-1.07)	0.465	1.02 (0.85-1.23)	0.804	0.477	NA	threosphingosine	0.98 (0.88-1.09)	0.756	1.05 (0.87-1.26)	0.588	0.656
HMDB00301	urocanic acid	0.98 (0.89-1.08)	0.7	1.04 (0.87-1.23)	0.684	0.718	HMDB00301	urocanic acid	0.97 (0.88-1.07)	0.541	1.04 (0.87-1.24)	0.657	0.658
HMDB00092	dimethylglycine	1.05 (0.95-1.17)	0.321	1.04 (0.85-1.25)	0.728	0.875	HMDB00092	dimethylglycine	1.08 (0.97-1.2)	0.182	1.08 (0.88-1.31)	0.464	0.667
HMDB01906*	aminoisobutyric acid	1.12 (1-1.24)	0.04	1.02 (0.84-1.23)	0.861	0.794	HMDB01906*	aminoisobutyric acid	1.11 (1-1.24)	0.049	1.02 (0.84-1.23)	0.842	0.673
HMDB11503	C16:0 LPE	1.05 (0.95-1.17)	0.361	1.04 (0.86-1.26)	0.687	0.784	HMDB11503	C16:0 LPE	1.02 (0.91-1.13)	0.783	0.99 (0.82-1.21)	0.942	0.675
NA	C20:4 carnitine	1.01 (0.91-1.12)	0.873	1.01 (0.84-1.22)	0.909	0.332	NA	C20:4 carnitine	1.03 (0.93-1.15)	0.586	1.02 (0.85-1.23)	0.829	0.676
NA	C34:1 DAG_or_TAG_fragment	0.92 (0.82-1.02)	0.113	0.94 (0.78-1.14)	0.555	0.796	NA	C34:1 DAG_or_TAG_fragment	0.92 (0.82-1.04)	0.185	1.02 (0.82-1.25)	0.887	0.676
HMDB04207	alpha-glutamyllysine	1.1 (0.98-1.23)	0.118	0.91 (0.74-1.13)	0.387	0.594	HMDB04207	alpha-glutamyllysine	1.1 (0.97-1.24)	0.126	0.92 (0.75-1.14)	0.449	0.685
HMDB01325	N6,N6,N6-trimethyllysine	0.98 (0.88-1.08)	0.683	1.04 (0.85-1.25)	0.713	0.776	HMDB01325	N6,N6,N6-trimethyllysine	0.97 (0.87-1.07)	0.52	1.04 (0.85-1.25)	0.704	0.685
HMDB00289	uric acid	0.95 (0.86-1.05)	0.309	1.07 (0.89-1.57)	0.606	0.754	HMDB00289	uric acid	0.96 (0.87-1.06)	0.412	1.17 (0.92-1.91)	0.422	0.686
HMDB01847	caffeine	0.95 (0.85-1.06)	0.349	0.88 (0.73-1.08)	0.207	0.8	HMDB01847	caffeine	0.94 (0.84-1.05)	0.251	0.87 (0.71-1.06)	0.154	0.69
HMDB07883*	C34:4 PC	0.9 (0.81-1.01)	0.068	0.97 (0.8-1.18)	0.784	0.993	HMDB07883*	C34:4 PC	0.89 (0.79-0.99)	0.039	1.01 (0.83-1.24)	0.926	0.703
HMDB06344	phenylacetylglutamine	0.88 (0.8-0.97)	0.013	0.97 (0.81-1.18)	0.757	0.731	HMDB06344	phenylacetylglutamine	0.88 (0.8-0.98)	0.019	0.96 (0.8-1.17)	0.694	0.712
HMDB00289	urate	0.95 (0.86-1.04)	0.254	1.08 (0.89-1.57)	0.597	0.786	HMDB00289	urate	0.95 (0.86-1.05)	0.341	1.18 (0.92-1.9)	0.399	0.713
HMDB10384	C18:0 LPC	1.02 (0.92-1.14)	0.671	1.13 (0.93-1.36)	0.218	0.408	HMDB10384	C18:0 LPC	1.03 (0.92-1.14)	0.635	1.12 (0.93-1.36)	0.242	0.717
HMDB00725	hydroxyproline	0.93 (0.84-1.03)	0.176	0.99 (0.81-1.19)	0.89	0.846	HMDB00725	hydroxyproline	0.94 (0.84-1.04)	0.246	1 (0.82-1.2)	0.968	0.723
HMDB02815*	C18:1 LPC	1.04 (0.94-1.16)	0.409	1.14 (0.95-1.38)	0.158	0.596	HMDB02815*	C18:1 LPC	1.03 (0.92-1.15)	0.596	1.1 (0.91-1.34)	0.324	0.732
HMDB04824	N2,N2-dimethylguanosine	0.93 (0.84-1.03)	0.188	0.97 (0.81-1.18)	0.791	0.771	HMDB04824	N2,N2-dimethylguanosine	0.94 (0.84-1.05)	0.254	1.03 (0.85-1.26)	0.75	0.736
HMDB04030	21-deoxycortisol	0.99 (0.89-1.11)	0.888	0.99 (0.81-1.22)	0.919	0.756	HMDB04030	21-deoxycortisol	0.98 (0.87-1.1)	0.753	0.96 (0.78-1.19)	0.69	0.741
HMDB10391*	C20:1 LPC	1.13 (1.01-1.26)	0.029	1.21 (0.99-1.47)	0.064	0.515	HMDB10391*	C20:1 LPC	1.13 (1.01-1.27)	0.038	1.15 (0.94-1.41)	0.188	0.744
HMDB11507*	C18:2 LPE	1.01 (0.9-1.12)	0.9	1.14 (0.94-1.38)	0.194	0.596	HMDB11507*	C18:2 LPE	1 (0.89-1.11)	0.96	1.11 (0.91-1.35)	0.314	0.748
HMDB00884	ribothymidine	0.98 (0.88-1.08)	0.663	1.09 (0.9-1.34)	0.393	0.852	HMDB00884	ribothymidine	0.96 (0.86-1.06)	0.407	1.06 (0.88-1.3)	0.551	0.756
HMDB00714	hippurate	1.02 (0.92-1.13)	0.689	1.07 (0.88-1.29)	0.5	0.438	HMDB00714	hippurate	1.01 (0.91-1.12)	0.847	1.04 (0.85-1.26)	0.709	0.756
NA	C34:2 DAG_or_TAG_fragment	0.93 (0.84-1.04)	0.208	0.99 (0.81-1.19)	0.881	0.962	NA	C34:2 DAG_or_TAG_fragment	0.95 (0.85-1.07)	0.425	1.08 (0.87-1.33)	0.489	0.757
HMDB00791	C8 carnitine	1 (0.89-1.11)	0.953	0.9 (0.74-1.1)	0.295	0.658	HMDB00791	C8 carnitine	1 (0.89-1.12)	0.971	0.92 (0.76-1.11)	0.375	0.758
HMDB00062	carnitine	0.94 (0.85-1.04)	0.222	1.01 (0.84-1.24)	0.881	0.588	HMDB00062	carnitine	0.95 (0.85-1.05)	0.287	1.05 (0.87-1.28)	0.649	0.758
HMDB07973*	C34:2 PC	1 (0.9-1.11)	0.955	0.96 (0.8-1.16)	0.676	0.743	HMDB07973*	C34:2 PC	0.98 (0.88-1.09)	0.758	0.96 (0.8-1.16)	0.649	0.758
HMDB10386*	C18:2 LPC	1.05 (0.95-1.17)	0.348	1.14 (0.94-1.38)	0.184	0.539	HMDB10386*	C18:2 LPC	1.03 (0.92-1.16)	0.606	1.08 (0.88-1.32)	0.489	0.762
HMDB13713	N-acetyltryptophan	1 (0.9-1.11)	0.978	1.05 (0.86-1.26)	0.647	0.86	HMDB13713	N-acetyltryptophan	0.97 (0.87-1.08)	0.601	1.02 (0.84-1.24)	0.843	0.763
HMDB01886	3-methylxanthine	1.02 (0.91-1.13)	0.78	1.03 (0.85-1.26)	0.773	0.846	HMDB01886	3-methylxanthine	1.02 (0.92-1.14)	0.675	1.02 (0.84-1.25)	0.864	0.764
HMDB08928*	C34:2 PE	0.93 (0.84-1.04)	0.219	0.98 (0.8-1.2)	0.84	0.914	HMDB08928*	C34:2 PE	0.92 (0.82-1.04)	0.184	1 (0.82-1.24)	0.977	0.771
HMDB08925*	C34:0 PE	0.96 (0.87-1.07)	0.458	1.02 (0.85-1.23)	0.834	0.611	HMDB08925*	C34:0 PE	0.94 (0.84-1.04)	0.221	1.02 (0.85-1.24)	0.819	0.776
HMDB10383*	C16:1 LPC	0.98 (0.88-1.08)	0.647	1.07 (0.88-1.29)	0.516	0.706	HMDB10383*	C16:1 LPC	0.97 (0.88-1.08)	0.614	1.08 (0.89-1.31)	0.449	0.78
HMDB13238	C7 carnitine	1.03 (0.93-1.15)	0.58	1.03 (0.86-1.25)	0.763	0.687	HMDB13238	C7 carnitine	1.03 (0.92-1.14)	0.648	1.05 (0.87-1.28)	0.646	0.781
HMDB11229*	C38:7 PC plasmalogen	1.03 (0.93-1.15)	0.546	0.94 (0.78-1.14)	0.542	0.7	HMDB11229*	C38:7 PC plasmalogen	1 (0.89-1.11)	0.954	0.91 (0.74-1.11)	0.338	0.786
HMDB01563	1-methylguanosine	0.96 (0.87-1.06)	0.457	0.97 (0.8-1.15)	0.717	0.889	HMDB01563	1-methylguanosine	0.97 (0.87-1.08)	0.581	1.01 (0.84-1.19)	0.954	0.789
HMDB00172	isoleucine	0.94 (0.85-1.04)	0.252	0.91 (0.75-1.1)	0.325	0.656	HMDB00172	isoleucine	0.96 (0.86-1.07)	0.468	0.97 (0.79-1.18)	0.751	0.79
HMDB00158	tyrosine	0.97 (0.88-1.08)	0.593	1.01 (0.83-1.22)	0.929	0.887	HMDB00158	tyrosine	0.97 (0.87-1.09)	0.637	1.07 (0.87-1.31)	0.523	0.792
HMDB05923	N4-acetylcytidine	0.97 (0.87-1.07)	0.503	0.98 (0.8-1.18)	0.797	0.854	HMDB05923	N4-acetylcytidine	0.98 (0.88-1.09)	0.715	1.03 (0.85-1.26)	0.739	0.795

HMDB00767	pseudouridine	0.93 (0.84-1.03)	0.183	1.04 (0.86-1.26)	0.682	0.965
HMDB00883	valine	0.93 (0.84-1.03)	0.192	0.88 (0.73-1.07)	0.203	0.814
HMDB10395	C20:4 LPC	1.01 (0.91-1.12)	0.897	1.03 (0.86-1.25)	0.727	0.739
HMDB01991	7-methylxanthine	1.02 (0.92-1.13)	0.732	1.07 (0.88-1.31)	0.499	0.859
HMDB00991*	2-aminooctanoic acid	0.98 (0.89-1.09)	0.762	0.99 (0.82-1.2)	0.939	0.661
HMDB00630	cytosine	0.97 (0.88-1.07)	0.523	0.98 (0.81-1.19)	0.8	0.881
NA	C46:2 TAG	0.88 (0.79-0.98)	0.018	0.95 (0.79-1.15)	0.618	0.842
HMDB00679	homocitrulline	1 (0.9-1.11)	0.957	0.92 (0.77-1.11)	0.369	0.786
NA	C46:3 TAG	0.88 (0.79-0.98)	0.024	0.97 (0.8-1.17)	0.74	0.788
HMDB11526	C22:6 LPE	1.04 (0.93-1.16)	0.476	1.05 (0.87-1.28)	0.605	0.9
HMDB02013	C4 carnitine	0.93 (0.84-1.04)	0.197	0.98 (0.81-1.18)	0.805	0.828
Internal Standard	valine-d8	1.04 (0.94-1.16)	0.441	0.92 (0.79-1.08)	0.275	0.544
HMDB00705	C6 carnitine	0.96 (0.86-1.07)	0.484	0.96 (0.79-1.17)	0.717	0.962
HMDB10169	C16:0 SM	1.08 (0.98-1.2)	0.138	0.95 (0.79-1.14)	0.545	0.934
HMDB00688	C5 carnitine	0.95 (0.86-1.06)	0.377	1.09 (0.9-1.31)	0.372	0.844
HMDB00716	pipecolic acid	0.99 (0.89-1.1)	0.86	1.01 (0.82-1.23)	0.933	0.834
HMDB11506*	C18:1 LPE	1.01 (0.91-1.13)	0.819	1.1 (0.91-1.34)	0.335	0.857
HMDB00026	N-carbamoyl-beta-alanine	0.94 (0.85-1.03)	0.198	1.02 (0.84-1.23)	0.849	0.654
HMDB11394*	C40:7 PE plasmalogen	1.09 (0.99-1.21)	0.092	1.03 (0.85-1.24)	0.784	0.999
HMDB13127	C4-OH carnitine	1.06 (0.95-1.18)	0.306	1.14 (0.94-1.4)	0.189	0.843
HMDB04400	5-acetylamino-6-amino-3-methylu	0.91 (0.82-1.01)	0.083	0.92 (0.77-1.13)	0.391	0.948
HMDB00162	proline	0.98 (0.88-1.09)	0.709	1 (0.83-1.22)	0.96	0.988
HMDB05066	C14 carnitine	1.02 (0.92-1.13)	0.745	1.05 (0.87-1.27)	0.627	0.764
HMDB01396	glycocholate	0.99 (0.89-1.1)	0.86	1.03 (0.85-1.26)	0.743	0.887
HMDB04827	proline betaine	0.96 (0.86-1.06)	0.391	1 (0.83-1.21)	0.984	0.605
HMDB59824	4-hydroxy-3-methylacetophenon	1 (0.91-1.11)	0.926	0.99 (0.83-1.2)	0.913	0.848
HMDB10404	C22:6 LPC	1.03 (0.93-1.14)	0.611	1.11 (0.92-1.35)	0.261	0.852
HMDB11478*	C18:3 LPE	0.96 (0.87-1.06)	0.438	1.15 (0.94-1.44)	0.206	0.717
HMDB10382	C16:0 LPC	1.02 (0.92-1.13)	0.695	1.07 (0.89-1.28)	0.494	0.588
HMDB01257	spermidine	1.1 (0.99-1.22)	0.073	1.01 (0.83-1.23)	0.908	0.884
HMDB11517	C20:4 LPE	1.01 (0.91-1.13)	0.79	1.04 (0.86-1.26)	0.703	0.926
HMDB07869*	C30:0 PC	0.93 (0.84-1.04)	0.198	0.98 (0.81-1.19)	0.855	0.732
HMDB00063	cortisol	0.98 (0.87-1.09)	0.683	0.98 (0.81-1.21)	0.86	0.921
NA	C44:2 TAG	0.89 (0.8-0.99)	0.027	0.97 (0.8-1.17)	0.726	0.877
HMDB10403	C22:5 LPC	0.95 (0.86-1.06)	0.356	1.03 (0.86-1.25)	0.73	0.936
HMDB00991*	2-aminooctanoic acid	1.06 (0.96-1.18)	0.255	1.03 (0.85-1.25)	0.783	0.781
HMDB00033	carnosine	0.96 (0.87-1.07)	0.447	0.88 (0.74-1.07)	0.188	0.773
HMDB11103	1,7-dimethyluric acid	0.9 (0.81-1)	0.05	0.92 (0.77-1.12)	0.389	0.974

HMDB00767	pseudouridine	0.94 (0.84-1.05)	0.252	1.09 (0.9-1.32)	0.381	0.802
HMDB00883	valine	0.95 (0.85-1.06)	0.337	0.95 (0.77-1.16)	0.613	0.803
HMDB10395	C20:4 LPC	1.01 (0.91-1.12)	0.898	1.02 (0.84-1.24)	0.846	0.806
HMDB01991	7-methylxanthine	1.04 (0.93-1.15)	0.486	1.07 (0.88-1.31)	0.529	0.816
HMDB00991*	2-aminooctanoic acid	0.99 (0.9-1.1)	0.872	0.98 (0.81-1.18)	0.809	0.819
HMDB00630	cytosine	0.96 (0.87-1.06)	0.423	0.96 (0.8-1.18)	0.717	0.829
NA	C46:2 TAG	0.87 (0.78-0.97)	0.015	0.98 (0.81-1.2)	0.866	0.835
HMDB00679	homocitrulline	0.98 (0.88-1.09)	0.722	0.91 (0.76-1.11)	0.333	0.838
NA	C46:3 TAG	0.88 (0.79-0.98)	0.025	1 (0.82-1.22)	0.961	0.845
HMDB11526	C22:6 LPE	1.01 (0.91-1.13)	0.848	1.03 (0.85-1.26)	0.736	0.864
HMDB02013	C4 carnitine	0.93 (0.83-1.03)	0.17	1 (0.82-1.21)	0.982	0.873
Internal Standard	valine-d8	1.05 (0.94-1.16)	0.4	0.94 (0.81-1.11)	0.425	0.877
HMDB00705	C6 carnitine	0.97 (0.87-1.08)	0.583	1 (0.82-1.21)	0.989	0.878
HMDB10169	C16:0 SM	1.07 (0.97-1.2)	0.19	0.94 (0.78-1.13)	0.486	0.882
HMDB00688	C5 carnitine	0.96 (0.86-1.07)	0.496	1.15 (0.95-1.4)	0.144	0.882
HMDB00716	pipecolic acid	0.97 (0.87-1.09)	0.641	1.01 (0.82-1.24)	0.911	0.889
HMDB11506*	C18:1 LPE	1 (0.9-1.12)	0.964	1.07 (0.88-1.31)	0.481	0.892
HMDB00026	N-carbamoyl-beta-alanine	0.93 (0.84-1.03)	0.183	1.03 (0.85-1.25)	0.75	0.906
HMDB11394*	C40:7 PE plasmalogen	1.07 (0.96-1.2)	0.192	0.99 (0.82-1.2)	0.921	0.912
HMDB13127	C4-OH carnitine	1.09 (0.97-1.22)	0.135	1.21 (0.99-1.49)	0.074	0.935
HMDB04400	5-acetylamino-6-amino-3-meth	0.92 (0.83-1.03)	0.156	0.91 (0.76-1.11)	0.334	0.936
HMDB00162	proline	0.99 (0.89-1.11)	0.914	1.05 (0.87-1.28)	0.598	0.939
HMDB05066	C14 carnitine	1.02 (0.92-1.14)	0.672	1.07 (0.88-1.3)	0.495	0.939
HMDB01396	glycocholate	1 (0.9-1.12)	0.949	1.07 (0.88-1.32)	0.493	0.941
HMDB04827	proline betaine	0.94 (0.85-1.05)	0.267	0.99 (0.82-1.2)	0.92	0.944
HMDB59824	4-hydroxy-3-methylacetophen	1.01 (0.91-1.12)	0.901	1.01 (0.85-1.23)	0.89	0.947
HMDB10404	C22:6 LPC	1 (0.89-1.11)	0.941	1.08 (0.89-1.32)	0.444	0.95
HMDB11478*	C18:3 LPE	0.95 (0.85-1.05)	0.274	1.12 (0.92-1.4)	0.292	0.951
HMDB10382	C16:0 LPC	1.01 (0.91-1.12)	0.892	1.05 (0.87-1.27)	0.603	0.952
HMDB01257	spermidine	1.1 (0.99-1.22)	0.089	1.03 (0.84-1.25)	0.776	0.959
HMDB11517	C20:4 LPE	1.02 (0.92-1.14)	0.712	1.03 (0.85-1.26)	0.739	0.961
HMDB07869*	C30:0 PC	0.9 (0.8-1)	0.055	0.98 (0.81-1.19)	0.859	0.965
HMDB00063	cortisol	0.97 (0.87-1.1)	0.663	0.96 (0.79-1.19)	0.727	0.969
NA	C44:2 TAG	0.88 (0.78-0.98)	0.022	0.99 (0.81-1.21)	0.956	0.984
HMDB10403	C22:5 LPC	0.94 (0.85-1.05)	0.292	1.01 (0.83-1.23)	0.909	0.985
HMDB00991*	2-aminooctanoic acid	1.06 (0.96-1.18)	0.253	1 (0.83-1.22)	0.986	0.991
HMDB00033	carnosine	0.95 (0.86-1.06)	0.366	0.9 (0.75-1.09)	0.289	0.994
HMDB11103	1,7-dimethyluric acid	0.9 (0.81-1.01)	0.06	0.91 (0.76-1.11)	0.327	0.999

Supplemental Table 4. : Odds ratios (OR) of breast cancer and 95% confidence intervals (CI) per 1 SD increase for all metabolites, by BMI categories (normal BMI: BMI<25, high BMI: BMI≥25).

Simple model: adjusts for matching factors including menopause status at blood draw, time of blood draw, date/season of blood draw, luteal day at blood draw, fasting status at blood draw, menopausal status at diagnosis and race.

Adjusted model: in addition to matching factors, this model adjusts for BMI at age 18, weight change between age 18 and time of blood draw, age at menarche, parity and age at first birth, family history of breast cancer, personal history of benign breast disease, physical activity, alcohol consumption, exogenous hormone use, breast feeding history.

Simple Model						
		High BMI		Normal BMI		p.het
HMDB_ID	METABOLITE	OR (95% CI)	PVAL	OR (95% CI)	PVAL	
NA	C36:4 DAG_or_TAG_fragment	0.89 (0.77-1.01)	0.077	1.18 (1.04-1.33)	0.008	0.001
HMDB04030	21-deoxycortisol	1.08 (0.95-1.24)	0.259	0.91 (0.79-1.05)	0.191	0.008
HMDB00187	serine	1.17 (1.02-1.34)	0.027	0.93 (0.83-1.05)	0.242	0.022
NA	C34:1 DAG_or_TAG_fragment	0.88 (0.77-1.01)	0.062	1.16 (1.03-1.31)	0.019	0.002
HMDB04827	proline betaine	0.83 (0.73-0.95)	0.007	1.04 (0.92-1.17)	0.538	0.018
HMDB09012*	C40:6 PE	0.87 (0.75-1)	0.047	1.1 (0.96-1.25)	0.168	0.011
NA	C38:5 DAG_or_TAG_fragment	0.88 (0.77-1.01)	0.076	1.13 (0.99-1.3)	0.077	0.009
NA	C36:3 DAG_or_TAG_fragment	0.92 (0.8-1.05)	0.201	1.15 (1.01-1.31)	0.031	0.012
HMDB00679	homocitrulline	0.87 (0.76-0.99)	0.034	1.08 (0.96-1.22)	0.203	0.032
NA	DMGV	0.78 (0.67-0.91)	0.001	0.93 (0.82-1.05)	0.246	0.044
HMDB01859	acetaminophen	1.08 (0.95-1.23)	0.239	0.9 (0.8-1.01)	0.074	0.032
HMDB13325	C10:2 carnitine	0.83 (0.71-0.96)	0.016	1.01 (0.9-1.13)	0.858	0.033
NA	C34:2 DAG_or_TAG_fragment	0.9 (0.79-1.04)	0.148	1.12 (0.99-1.27)	0.074	0.016
HMDB00714	hippurate	1.13 (0.99-1.29)	0.073	0.96 (0.85-1.07)	0.445	0.032
NA	N-methylproline	0.84 (0.73-0.96)	0.011	1.04 (0.93-1.16)	0.529	0.025
HMDB00128	guanidinoacetic acid	1.15 (1.01-1.32)	0.041	0.97 (0.86-1.09)	0.63	0.072
HMDB09003*	C38:4 PE	0.87 (0.76-1)	0.052	1.07 (0.94-1.23)	0.285	0.034
HMDB07874*	C32:2 PC	0.93 (0.8-1.07)	0.302	1.08 (0.95-1.21)	0.234	0.06
HMDB09102*	C38:6 PE	0.93 (0.8-1.07)	0.294	1.1 (0.97-1.25)	0.12	0.055
HMDB00883	valine	0.82 (0.71-0.94)	0.005	1.01 (0.9-1.15)	0.816	0.018
NA	C34:2 DAG_or_TAG_fragment	0.9 (0.79-1.03)	0.114	1.1 (0.96-1.27)	0.169	0.022
HMDB00812	N-acetylaspartic acid	1.11 (0.96-1.27)	0.153	0.93 (0.83-1.05)	0.223	0.088
HMDB00884	ribothymidine	1.11 (0.97-1.29)	0.136	0.95 (0.84-1.06)	0.358	0.054
HMDB00687	leucine	0.83 (0.72-0.95)	0.008	1.02 (0.9-1.15)	0.769	0.025
HMDB00123	glycine	1.08 (0.94-1.23)	0.273	0.95 (0.84-1.07)	0.377	0.099
HMDB11526	C22:6 LPE	1.01 (0.87-1.16)	0.904	1.14 (1.01-1.28)	0.038	0.104
NA	C34:3 DAG_or_TAG_fragment	0.9 (0.79-1.04)	0.152	1.1 (0.96-1.25)	0.18	0.03
HMDB08994*	C36:2 PE	0.87 (0.76-1)	0.05	1.05 (0.92-1.19)	0.495	0.049
HMDB07883*	C34:4 PC	0.88 (0.76-1.02)	0.083	1.01 (0.89-1.14)	0.926	0.087
HMDB00631*	glycodeoxycholate/glycochenodeo	1.1 (0.95-1.27)	0.201	0.93 (0.82-1.06)	0.273	0.194
HMDB02815*	C18:1 LPC	1.11 (0.97-1.28)	0.131	0.96 (0.85-1.08)	0.467	0.079
HMDB00289	uric acid	0.72 (0.51-1.01)	0.061	1 (0.91-1.1)	0.968	0.011
HMDB00289	urate	0.72 (0.51-1.01)	0.056	1 (0.91-1.09)	0.941	0.011
HMDB11512*	C20:1 LPE	1.07 (0.94-1.21)	0.34	0.92 (0.81-1.03)	0.155	0.12
HMDB00063	cortisol	1.05 (0.91-1.21)	0.512	0.97 (0.85-1.11)	0.692	0.153
HMDB01906*	aminoisobutyric acid	1.12 (0.98-1.28)	0.098	1.03 (0.92-1.16)	0.622	0.339
HMDB32055	N-acetylhistidine	1.04 (0.9-1.19)	0.584	0.95 (0.84-1.07)	0.39	0.27
HMDB01008	biliverdin	0.96 (0.78-1.19)	0.739	1.07 (0.97-1.2)	0.177	0.326
HMDB02802	cortisone	1.02 (0.89-1.18)	0.773	0.96 (0.85-1.09)	0.533	0.284
HMDB00159	phenylalanine	0.89 (0.77-1.03)	0.116	1.08 (0.96-1.21)	0.199	0.028
HMDB01565	phosphocholine	1.13 (0.99-1.3)	0.069	1.03 (0.91-1.17)	0.591	0.136
HMDB11441*	C36:3 PE plasmalogen	1.08 (0.95-1.23)	0.254	0.98 (0.87-1.1)	0.705	0.147
HMDB00630	cytosine	1.01 (0.89-1.16)	0.838	0.94 (0.83-1.05)	0.278	0.299
HMDB00716	pipecolic acid	0.89 (0.77-1.03)	0.112	1.04 (0.92-1.17)	0.526	0.116
HMDB00161	alanine	0.88 (0.76-1.02)	0.093	1.02 (0.91-1.16)	0.688	0.048
HMDB29377	piperine	0.76 (0.66-0.89)	0	0.9 (0.81-1.01)	0.071	0.122
HMDB04207	alpha-glutamyllysine	1.08 (0.93-1.26)	0.301	0.99 (0.87-1.13)	0.885	0.621

Adjusted Model						
		High BMI		Normal BMI		p.het
HMDB_ID	METABOLITE	OR (95% CI)	PVAL	OR (95% CI)	PVAL	
NA	C36:4 DAG_or_TAG_fragment	0.92 (0.8-1.06)	0.26	1.21 (1.06-1.37)	0.004	0.005
HMDB04030	21-deoxycortisol	1.08 (0.94-1.25)	0.278	0.89 (0.77-1.03)	0.109	0.007
HMDB00187	serine	1.19 (1.04-1.38)	0.013	0.93 (0.83-1.05)	0.249	0.008
NA	C34:1 DAG_or_TAG_fragment	0.92 (0.79-1.06)	0.253	1.18 (1.04-1.34)	0.01	0.009
HMDB04827	proline betaine	0.82 (0.72-0.94)	0.005	1.04 (0.92-1.17)	0.557	0.017
HMDB09012*	C40:6 PE	0.87 (0.75-1.01)	0.066	1.09 (0.96-1.25)	0.197	0.023
NA	C38:5 DAG_or_TAG_fragment	0.92 (0.79-1.06)	0.242	1.12 (0.98-1.29)	0.102	0.028
NA	C36:3 DAG_or_TAG_fragment	0.97 (0.84-1.11)	0.627	1.18 (1.03-1.35)	0.017	0.033
HMDB00679	homocitrulline	0.86 (0.75-0.99)	0.031	1.08 (0.96-1.23)	0.209	0.036
NA	DMGV	0.82 (0.7-0.97)	0.019	0.94 (0.83-1.06)	0.32	0.038
HMDB01859	acetaminophen	1.05 (0.92-1.21)	0.454	0.89 (0.79-1)	0.059	0.042
HMDB13325	C10:2 carnitine	0.83 (0.71-0.98)	0.024	1.01 (0.9-1.13)	0.9	0.05
NA	C34:2 DAG_or_TAG_fragment	0.94 (0.81-1.09)	0.416	1.12 (0.99-1.27)	0.074	0.051
HMDB00714	hippurate	1.11 (0.96-1.28)	0.155	0.95 (0.84-1.07)	0.427	0.053
NA	N-methylproline	0.86 (0.74-0.99)	0.031	1.04 (0.93-1.17)	0.514	0.056
HMDB00128	guanidinoacetic acid	1.14 (0.99-1.31)	0.08	0.98 (0.87-1.1)	0.722	0.065
HMDB09003*	C38:4 PE	0.9 (0.78-1.03)	0.136	1.1 (0.96-1.26)	0.185	0.07
HMDB07874*	C32:2 PC	0.92 (0.79-1.06)	0.249	1.06 (0.94-1.2)	0.361	0.074
HMDB09102*	C38:6 PE	0.91 (0.78-1.05)	0.205	1.1 (0.97-1.25)	0.153	0.077
HMDB00883	valine	0.86 (0.74-1)	0.046	1 (0.88-1.13)	0.961	0.078
NA	C34:2 DAG_or_TAG_fragment	0.94 (0.82-1.09)	0.421	1.1 (0.95-1.27)	0.201	0.088
HMDB00812	N-acetylaspartic acid	1.1 (0.95-1.28)	0.193	0.92 (0.82-1.04)	0.199	0.09
HMDB00884	ribothymidine	1.09 (0.94-1.26)	0.266	0.93 (0.83-1.05)	0.253	0.093
HMDB00687	leucine	0.87 (0.75-1.01)	0.067	1.01 (0.89-1.14)	0.865	0.097
HMDB00123	glycine	1.07 (0.93-1.23)	0.355	0.96 (0.84-1.08)	0.485	0.101
HMDB11526	C22:6 LPE	0.98 (0.85-1.14)	0.818	1.12 (0.99-1.27)	0.082	0.111
NA	C34:3 DAG_or_TAG_fragment	0.95 (0.82-1.1)	0.518	1.09 (0.95-1.25)	0.208	0.111
HMDB08994*	C36:2 PE	0.89 (0.77-1.03)	0.112	1.06 (0.93-1.22)	0.355	0.114
HMDB07883*	C34:4 PC	0.88 (0.75-1.02)	0.086	0.98 (0.86-1.12)	0.767	0.119
HMDB00631*	glycodeoxycholate/glycochenodeo	1.14 (0.98-1.33)	0.098	0.94 (0.82-1.06)	0.31	0.125
HMDB02815*	C18:1 LPC	1.08 (0.94-1.25)	0.282	0.96 (0.85-1.08)	0.504	0.129
HMDB00289	uric acid	0.8 (0.55-1.16)	0.247	1 (0.91-1.1)	0.98	0.138
HMDB00289	urate	0.8 (0.56-1.15)	0.237	0.99 (0.9-1.09)	0.873	0.14
HMDB11512*	C20:1 LPE	1.04 (0.91-1.19)	0.605	0.91 (0.81-1.03)	0.143	0.154
HMDB00063	cortisol	1.06 (0.91-1.23)	0.466	0.96 (0.84-1.1)	0.569	0.156
HMDB01906*	aminoisobutyric acid	1.13 (0.99-1.3)	0.078	1.02 (0.9-1.15)	0.761	0.157
HMDB32055	N-acetylhistidine	1.06 (0.91-1.22)	0.458	0.95 (0.84-1.07)	0.421	0.158
HMDB01008	biliverdin	0.95 (0.76-1.18)	0.639	1.08 (0.98-1.21)	0.144	0.165
HMDB02802	cortisone	1.03 (0.89-1.19)	0.663	0.95 (0.84-1.08)	0.456	0.165
HMDB00159	phenylalanine	0.95 (0.82-1.11)	0.515	1.06 (0.94-1.19)	0.327	0.173
HMDB01565	phosphocholine	1.11 (0.97-1.29)	0.138	1.05 (0.93-1.19)	0.435	0.182
HMDB11441*	C36:3 PE plasmalogen	1.07 (0.93-1.22)	0.342	0.98 (0.87-1.11)	0.747	0.184
HMDB00630	cytosine	1.02 (0.89-1.17)	0.771	0.93 (0.83-1.05)	0.23	0.185
HMDB00716	pipecolic acid	0.89 (0.77-1.04)	0.147	1.03 (0.9-1.16)	0.7	0.197
HMDB00161	alanine	0.93 (0.79-1.09)	0.385	1.02 (0.9-1.15)	0.808	0.199
HMDB29377	piperine	0.74 (0.63-0.86)	0	0.88 (0.78-0.98)	0.025	0.202
HMDB04207	alpha-glutamyllysine	1.13 (0.97-1.32)	0.132	0.97 (0.85-1.1)	0.619	0.206

HMDB13238	C7 carnitine	1.12 (0.97-1.29)	0.136	0.98 (0.88-1.1)	0.735	0.32	HMDB13238	C7 carnitine	1.13 (0.97-1.31)	0.11	0.97 (0.86-1.08)	0.56	0.21
HMDB05862	2-methylguanosine	1.16 (0.99-1.38)	0.08	1.03 (0.92-1.15)	0.664	0.346	HMDB05862	2-methylguanosine	1.22 (1.02-1.47)	0.038	1.02 (0.9-1.14)	0.787	0.216
NA	C36:2 DAG_or_TAG_fragment	0.92 (0.81-1.05)	0.233	1.07 (0.93-1.22)	0.354	0.097	NA	C36:2 DAG_or_TAG_fragment	0.97 (0.85-1.12)	0.707	1.07 (0.93-1.22)	0.347	0.217
HMDB04193	N1-methyl-2-pyridone-5-carboxam	0.86 (0.74-1)	0.046	1.01 (0.9-1.13)	0.831	0.069	HMDB04193	N1-methyl-2-pyridone-5-carboxam	0.88 (0.76-1.02)	0.097	0.99 (0.88-1.11)	0.803	0.218
HMDB00562	creatinine	1 (0.87-1.14)	0.962	0.91 (0.8-1.02)	0.098	0.161	HMDB00562	creatinine	0.99 (0.86-1.14)	0.915	0.91 (0.8-1.02)	0.108	0.219
HMDB01563	1-methylguanosine	1.03 (0.89-1.2)	0.69	0.96 (0.85-1.07)	0.433	0.428	HMDB01563	1-methylguanosine	1.11 (0.95-1.3)	0.183	0.94 (0.84-1.06)	0.316	0.222
HMDB04400	5-acetylamino-6-amino-3-methylur	1 (0.86-1.17)	0.976	0.86 (0.77-0.96)	0.009	0.132	HMDB04400	5-acetylamino-6-amino-3-methylu	1.02 (0.87-1.19)	0.848	0.87 (0.77-0.98)	0.025	0.226
HMDB03357	N-acetylornithine	0.95 (0.83-1.08)	0.422	1.06 (0.94-1.19)	0.327	0.27	HMDB03357	N-acetylornithine	0.93 (0.81-1.07)	0.336	1.04 (0.93-1.17)	0.478	0.227
HMDB01886	3-methylxanthine	1.12 (0.96-1.3)	0.149	0.95 (0.85-1.06)	0.323	0.167	HMDB01886	3-methylxanthine	1.15 (0.98-1.34)	0.091	0.96 (0.85-1.07)	0.438	0.231
HMDB02366	C5:1 carnitine	0.92 (0.8-1.05)	0.227	1.05 (0.94-1.18)	0.395	0.189	HMDB02366	C5:1 carnitine	0.92 (0.8-1.06)	0.256	1.04 (0.92-1.17)	0.537	0.235
HMDB13130	C5-DC carnitine	0.92 (0.8-1.05)	0.208	1.02 (0.91-1.15)	0.758	0.317	HMDB13130	C5-DC carnitine	0.91 (0.79-1.05)	0.219	1.02 (0.9-1.15)	0.78	0.239
HMDB00641	glutamine	1.09 (0.94-1.26)	0.263	0.99 (0.88-1.11)	0.831	0.364	HMDB00641	glutamine	1.11 (0.95-1.29)	0.194	0.97 (0.86-1.09)	0.622	0.248
HMDB00688	C5 carnitine	0.89 (0.77-1.01)	0.082	1.01 (0.9-1.14)	0.817	0.137	HMDB00688	C5 carnitine	0.91 (0.79-1.05)	0.196	1.01 (0.89-1.14)	0.901	0.251
NA	glutamic acid amide	1.08 (0.93-1.26)	0.292	0.99 (0.88-1.11)	0.818	0.386	NA	glutamic acid amide	1.1 (0.95-1.29)	0.21	0.97 (0.86-1.09)	0.603	0.251
HMDB00172	isoleucine	0.85 (0.74-0.97)	0.019	0.99 (0.87-1.11)	0.824	0.075	HMDB00172	isoleucine	0.9 (0.77-1.04)	0.147	0.98 (0.86-1.11)	0.708	0.255
HMDB01396	glycocholate	1.04 (0.9-1.21)	0.584	0.97 (0.86-1.09)	0.624	0.554	HMDB01396	glycocholate	1.09 (0.93-1.28)	0.28	0.98 (0.86-1.1)	0.682	0.261
HMDB02820	methylimidazole acetic acid	0.97 (0.85-1.11)	0.658	1.04 (0.92-1.17)	0.54	0.702	HMDB02820	methylimidazole acetic acid	0.94 (0.82-1.08)	0.404	1.04 (0.93-1.18)	0.475	0.268
HMDB01847	caffeine	1.04 (0.89-1.21)	0.633	0.89 (0.79-1)	0.046	0.247	HMDB01847	caffeine	1.02 (0.87-1.2)	0.787	0.89 (0.78-1)	0.049	0.271
HMDB08937*	C36:4 PE	0.91 (0.79-1.04)	0.167	1.04 (0.92-1.19)	0.521	0.137	HMDB08937*	C36:4 PE	0.92 (0.79-1.06)	0.23	1.05 (0.92-1.21)	0.478	0.273
HMDB11343*	C34:3 PE plasmalogen	1.05 (0.92-1.19)	0.49	0.99 (0.88-1.11)	0.864	0.344	HMDB11343*	C34:3 PE plasmalogen	1.04 (0.91-1.19)	0.556	0.98 (0.87-1.11)	0.77	0.276
HMDB05923	N4-acetylcytidine	0.99 (0.87-1.13)	0.856	0.94 (0.83-1.06)	0.288	0.493	HMDB05923	N4-acetylcytidine	1.05 (0.91-1.21)	0.528	0.94 (0.83-1.06)	0.303	0.28
HMDB11103	1,7-dimethyluric acid	1 (0.86-1.17)	0.95	0.87 (0.77-0.97)	0.012	0.215	HMDB11103	1,7-dimethyluric acid	1.01 (0.86-1.18)	0.948	0.87 (0.77-0.98)	0.021	0.281
HMDB10391*	C20:1 LPC	1.18 (1.02-1.37)	0.03	1.04 (0.93-1.17)	0.456	0.173	HMDB10391*	C20:1 LPC	1.16 (1-1.36)	0.053	1.06 (0.94-1.19)	0.363	0.281
HMDB00904	citrulline	1.03 (0.9-1.18)	0.664	0.97 (0.87-1.1)	0.671	0.425	HMDB00904	citrulline	1.05 (0.91-1.22)	0.484	0.98 (0.87-1.11)	0.804	0.283
HMDB08928*	C34:2 PE	0.91 (0.8-1.04)	0.168	1.04 (0.92-1.19)	0.514	0.124	HMDB08928*	C34:2 PE	0.92 (0.8-1.06)	0.27	1.05 (0.92-1.21)	0.457	0.283
HMDB32390	2-methyl-4,5-benzoxazole	0.87 (0.76-1)	0.049	0.95 (0.85-1.07)	0.409	0.448	HMDB32390	2-methyl-4,5-benzoxazole	0.85 (0.73-0.98)	0.022	0.97 (0.86-1.09)	0.632	0.288
HMDB00062	carnitine	0.88 (0.76-1.01)	0.077	1.02 (0.91-1.14)	0.769	0.123	HMDB00062	carnitine	0.91 (0.78-1.06)	0.212	1 (0.89-1.12)	0.994	0.288
HMDB00177	histidine	0.94 (0.82-1.09)	0.405	1.05 (0.94-1.18)	0.393	0.226	HMDB00177	histidine	0.95 (0.82-1.1)	0.479	1.04 (0.92-1.17)	0.52	0.291
HMDB13631	oleoyl glycine	1.1 (0.95-1.27)	0.202	1 (0.88-1.13)	0.992	0.508	HMDB13631	oleoyl glycine	1.12 (0.97-1.3)	0.117	0.99 (0.87-1.12)	0.867	0.293
HMDB11420*	C38:7 PE plasmalogen	1.02 (0.88-1.18)	0.819	1.11 (0.99-1.24)	0.078	0.295	HMDB11420*	C38:7 PE plasmalogen	0.98 (0.83-1.14)	0.763	1.08 (0.96-1.21)	0.224	0.298
HMDB10379	C14:0 LPC	0.95 (0.83-1.09)	0.465	1.04 (0.92-1.16)	0.558	0.192	HMDB10379	C14:0 LPC	0.94 (0.82-1.09)	0.435	1.01 (0.89-1.14)	0.887	0.301
HMDB15168	cerulenin	0.91 (0.8-1.04)	0.187	0.99 (0.88-1.12)	0.893	0.315	HMDB15168	cerulenin	0.92 (0.8-1.05)	0.21	0.98 (0.87-1.11)	0.786	0.302
HMDB00162	proline	0.92 (0.79-1.06)	0.23	1.02 (0.91-1.15)	0.711	0.184	HMDB00162	proline	0.98 (0.84-1.14)	0.771	1.01 (0.9-1.14)	0.821	0.308
HMDB02013	C4 carnitine	0.88 (0.77-1.01)	0.071	1.01 (0.9-1.13)	0.841	0.129	HMDB02013	C4 carnitine	0.91 (0.79-1.05)	0.207	0.99 (0.88-1.11)	0.872	0.309
NA	threosphingosine	1.02 (0.89-1.16)	0.82	0.95 (0.84-1.06)	0.359	0.454	NA	threosphingosine	1.07 (0.93-1.22)	0.352	0.94 (0.83-1.06)	0.286	0.324
NA	hydroxyectoine	1.03 (0.89-1.18)	0.712	0.98 (0.87-1.1)	0.691	0.46	NA	hydroxyectoine	1.05 (0.91-1.21)	0.533	0.99 (0.87-1.11)	0.835	0.329
HMDB13331	C14:2 carnitine	0.94 (0.81-1.09)	0.426	1.03 (0.92-1.16)	0.592	0.243	HMDB13331	C14:2 carnitine	0.95 (0.81-1.11)	0.524	1.03 (0.92-1.16)	0.591	0.329
HMDB00630*	cytosine_isomer	1.03 (0.9-1.19)	0.644	0.96 (0.85-1.08)	0.453	0.397	HMDB00630*	cytosine_isomer	1.04 (0.89-1.2)	0.64	0.93 (0.83-1.05)	0.265	0.333
HMDB34169	methyl N-methylanthranilate	1.08 (0.95-1.23)	0.26	0.99 (0.88-1.11)	0.862	0.244	HMDB34169	methyl N-methylanthranilate	1.08 (0.94-1.24)	0.27	0.99 (0.88-1.12)	0.887	0.339
HMDB00791	C8 carnitine	0.93 (0.8-1.09)	0.397	1.03 (0.92-1.16)	0.612	0.216	HMDB00791	C8 carnitine	0.93 (0.79-1.1)	0.406	1.02 (0.91-1.15)	0.691	0.341
HMDB02172	diacetylspermine	0.93 (0.81-1.07)	0.299	1.08 (0.97-1.22)	0.166	0.164	HMDB02172	diacetylspermine	0.97 (0.84-1.13)	0.726	1.09 (0.97-1.22)	0.159	0.342
HMDB00301	urocanic acid	1 (0.87-1.14)	0.988	0.95 (0.84-1.06)	0.364	0.623	HMDB00301	urocanic acid	1.01 (0.88-1.16)	0.834	0.93 (0.83-1.05)	0.226	0.348
NA	C34:1 DAG_or_TAG_fragment	0.91 (0.8-1.04)	0.151	1.03 (0.89-1.18)	0.7	0.14	NA	C34:1 DAG_or_TAG_fragment	0.95 (0.82-1.09)	0.459	1.01 (0.88-1.17)	0.87	0.35
HMDB08952*	C34:2 PE plasmalogen	1.05 (0.92-1.2)	0.435	1.01 (0.89-1.13)	0.921	0.4	HMDB08952*	C34:2 PE plasmalogen	1.05 (0.91-1.2)	0.508	1 (0.89-1.13)	1	0.354
HMDB11229*	C38:7 PC plasmalogen	1.01 (0.87-1.17)	0.897	1.07 (0.96-1.2)	0.223	0.509	HMDB11229*	C38:7 PC plasmalogen	0.96 (0.82-1.12)	0.601	1.06 (0.94-1.19)	0.366	0.355
NA	C36:2 PS plasmalogen	1.02 (0.89-1.17)	0.821	1.12 (1-1.26)	0.045	0.216	NA	C36:2 PS plasmalogen	1.03 (0.89-1.18)	0.721	1.12 (1-1.26)	0.06	0.357
HMDB02172	N1,N12-diacetylspermine	0.93 (0.81-1.07)	0.304	1.08 (0.96-1.21)	0.189	0.175	HMDB02172	N1,N12-diacetylspermine	0.97 (0.84-1.13)	0.726	1.09 (0.97-1.22)	0.164	0.358
HMDB12103	C22:0 SM	0.96 (0.83-1.1)	0.528	1.03 (0.92-1.17)	0.57	0.32	HMDB12103	C22:0 SM	0.97 (0.84-1.11)	0.633	1.03 (0.91-1.17)	0.605	0.361
HMDB00929	tryptophan	1.05 (0.92-1.21)	0.469	0.97 (0.86-1.09)	0.574	0.3	HMDB00929	tryptophan	1.06 (0.93-1.22)	0.385	0.95 (0.84-1.07)	0.426	0.363
HMDB04949	C16:0 Ceramide (d18:1)	1.05 (0.93-1.2)	0.411	0.98 (0.86-1.11)	0.768	0.544	HMDB04949	C16:0 Ceramide (d18:1)	1.07 (0.94-1.23)	0.297	0.97 (0.85-1.1)	0.665	0.365
HMDB11506*	C18:1 LPE	1.07 (0.92-1.24)	0.395	0.98 (0.87-1.11)	0.767	0.353	HMDB11506*	C18:1 LPE	1.06 (0.91-1.23)	0.455	0.99 (0.88-1.12)	0.92	0.373
HMDB00651	C10 carnitine	0.94 (0.81-1.1)	0.447	1.02 (0.91-1.15)	0.682	0.276	HMDB00651	C10 carnitine	0.93 (0.8-1.09)	0.402	1.02 (0.9-1.15)	0.759	0.378
HMDB13127	C4-OH carnitine	1.12 (0.97-1.31)	0.125	1.06 (0.94-1.19)	0.334	0.725	HMDB13127	C4-OH carnitine	1.22 (1.04-1.44)	0.016	1.07 (0.95-1.2)	0.274	0.386
HMDB11478*	C18:3 LPE	0.95 (0.82-1.09)	0.463	1.02 (0.91-1.15)	0.685	0.342	HMDB11478*	C18:3 LPE	0.93 (0.8-1.08)	0.336	1.02 (0.9-1.14)	0.794	0.403
HMDB00725	hydroxyproline	0.97 (0.84-1.11)	0.643	0.94 (0.84-1.06)	0.325	0.629	HMDB00725	hydroxyproline	1 (0.87-1.16)	0.966	0.95 (0.84-1.06)	0.363	0.411

HMDB10386*	C18:2 LPC	1.1 (0.95-1.28)	0.186	1.02 (0.9-1.16)	0.707	0.265	HMDB10386*	C18:2 LPC	1.05 (0.9-1.23)	0.516	1.03 (0.91-1.17)	0.663	0.412
NA	C16:1 SM	0.94 (0.81-1.09)	0.409	1.04 (0.92-1.18)	0.511	0.18	NA	C16:1 SM	0.97 (0.83-1.13)	0.68	1.04 (0.91-1.18)	0.561	0.422
HMDB00133	guanosine	1.05 (0.9-1.23)	0.507	0.97 (0.87-1.08)	0.584	0.389	HMDB00133	guanosine	1.05 (0.9-1.23)	0.55	0.97 (0.87-1.09)	0.628	0.423
HMDB13678	4-hydroxyhippurate	0.96 (0.84-1.09)	0.507	1.07 (0.96-1.21)	0.228	0.196	HMDB13678	4-hydroxyhippurate	0.96 (0.83-1.1)	0.515	1.06 (0.94-1.2)	0.328	0.438
HMDB00824	C3 carnitine	0.88 (0.76-1.01)	0.063	0.99 (0.88-1.11)	0.905	0.149	HMDB00824	C3 carnitine	0.91 (0.79-1.05)	0.203	0.98 (0.87-1.1)	0.742	0.454
HMDB12097	C14:0 SM	0.95 (0.82-1.1)	0.511	1.02 (0.91-1.15)	0.742	0.331	HMDB12097	C14:0 SM	0.94 (0.81-1.09)	0.42	1 (0.89-1.13)	0.95	0.457
HMDB10404	C22:6 LPC	1.04 (0.91-1.2)	0.546	1.09 (0.97-1.22)	0.158	0.563	HMDB10404	C22:6 LPC	1 (0.87-1.16)	0.991	1.06 (0.94-1.2)	0.317	0.459
HMDB11745	N-acetylmethionine	1.13 (0.99-1.29)	0.072	1.02 (0.91-1.15)	0.711	0.267	HMDB11745	N-acetylmethionine	1.14 (0.99-1.3)	0.065	1.03 (0.91-1.16)	0.65	0.469
HMDB05066	C14 carnitine	1.09 (0.94-1.26)	0.251	1.02 (0.91-1.14)	0.772	0.802	HMDB05066	C14 carnitine	1.12 (0.96-1.3)	0.137	1.01 (0.9-1.13)	0.913	0.481
HMDB00479	methylhistidine	0.96 (0.83-1.1)	0.56	1.02 (0.91-1.14)	0.703	0.63	HMDB00479	methylhistidine	0.95 (0.82-1.1)	0.512	1.01 (0.9-1.14)	0.827	0.486
HMDB04620	N-alpha-acetyarginine	1 (0.87-1.15)	0.976	0.93 (0.82-1.05)	0.225	0.547	HMDB04620	N-alpha-acetyarginine	1.01 (0.87-1.16)	0.939	0.93 (0.82-1.05)	0.221	0.493
HMDB00853	acetyl-galactosamine	1.01 (0.88-1.15)	0.909	1.03 (0.91-1.15)	0.678	0.884	HMDB00853	acetyl-galactosamine	1.08 (0.94-1.24)	0.288	1.02 (0.9-1.14)	0.807	0.494
HMDB00991*	2-aminooctanoic acid	0.98 (0.86-1.12)	0.781	1.06 (0.94-1.19)	0.371	0.602	HMDB00991*	2-aminooctanoic acid	0.99 (0.86-1.13)	0.851	1.07 (0.95-1.21)	0.263	0.499
HMDB11756	N-acetylleucine	1.01 (0.89-1.15)	0.899	1.01 (0.89-1.13)	0.926	0.911	HMDB11756	N-acetylleucine	1.05 (0.92-1.2)	0.495	1 (0.89-1.13)	0.967	0.52
HMDB06347	C26 carnitine	1.02 (0.89-1.16)	0.801	1.1 (0.98-1.23)	0.111	0.411	HMDB06347	C26 carnitine	1.02 (0.89-1.18)	0.725	1.11 (0.98-1.25)	0.092	0.522
HMDB13326	C12:1 carnitine	0.95 (0.81-1.11)	0.529	1.02 (0.91-1.14)	0.761	0.292	HMDB13326	C12:1 carnitine	0.97 (0.82-1.14)	0.703	1.02 (0.9-1.14)	0.797	0.524
HMDB01991	7-methylxanthine	1.08 (0.94-1.25)	0.276	0.97 (0.87-1.09)	0.625	0.344	HMDB01991	7-methylxanthine	1.11 (0.96-1.29)	0.174	0.99 (0.88-1.11)	0.839	0.531
HMDB00001	1-methylhistidine	0.96 (0.83-1.1)	0.537	1.02 (0.91-1.14)	0.793	0.648	HMDB00001	1-methylhistidine	0.95 (0.82-1.09)	0.46	1 (0.89-1.13)	0.941	0.536
HMDB00696	methionine	1.02 (0.88-1.19)	0.748	1.01 (0.9-1.13)	0.885	0.986	HMDB00696	methionine	1.06 (0.91-1.23)	0.47	0.99 (0.88-1.11)	0.88	0.537
HMDB11394*	C40:7 PE plasmalogen	1.09 (0.95-1.25)	0.237	1.13 (1.01-1.27)	0.037	0.712	HMDB11394*	C40:7 PE plasmalogen	1.05 (0.9-1.21)	0.556	1.13 (1-1.27)	0.049	0.541
HMDB00033	carnosine	1.02 (0.89-1.17)	0.773	0.95 (0.84-1.06)	0.325	0.47	HMDB00033	carnosine	1.03 (0.9-1.2)	0.647	0.94 (0.83-1.05)	0.257	0.55
HMDB10383*	C16:1 LPC	1 (0.87-1.15)	0.953	0.96 (0.86-1.08)	0.487	0.832	HMDB10383*	C16:1 LPC	1.01 (0.88-1.17)	0.883	0.95 (0.84-1.07)	0.39	0.551
HMDB00544	5-hydroxymethyl-4-methyluracil	0.99 (0.86-1.13)	0.865	0.93 (0.83-1.04)	0.215	0.574	HMDB00544	5-hydroxymethyl-4-methyluracil	1 (0.87-1.16)	0.966	0.92 (0.82-1.04)	0.174	0.558
HMDB10403	C22:5 LPC	1.01 (0.88-1.16)	0.929	0.93 (0.83-1.05)	0.247	0.398	HMDB10403	C22:5 LPC	0.98 (0.85-1.13)	0.785	0.93 (0.83-1.05)	0.258	0.56
HMDB13287	N6,N6-dimethyllysine	0.98 (0.85-1.12)	0.753	0.93 (0.83-1.04)	0.221	0.524	HMDB13287	N6,N6-dimethyllysine	0.99 (0.86-1.14)	0.931	0.94 (0.84-1.06)	0.303	0.563
HMDB11511	C20:0 LPE	1.11 (0.97-1.26)	0.135	1.03 (0.91-1.17)	0.651	0.475	HMDB11511	C20:0 LPE	1.08 (0.94-1.25)	0.265	1.04 (0.91-1.18)	0.563	0.567
HMDB00991*	2-aminooctanoic acid	1.01 (0.88-1.15)	0.91	0.95 (0.84-1.07)	0.386	0.494	HMDB00991*	2-aminooctanoic acid	0.99 (0.87-1.14)	0.923	0.96 (0.85-1.08)	0.526	0.573
HMDB02064	N-acetylputrescine	0.93 (0.81-1.07)	0.326	1.01 (0.89-1.14)	0.879	0.381	HMDB02064	N-acetylputrescine	0.93 (0.81-1.08)	0.358	1 (0.88-1.13)	0.98	0.576
HMDB00182	lysine	0.9 (0.78-1.05)	0.174	1.03 (0.91-1.18)	0.61	0.148	HMDB00182	lysine	0.95 (0.81-1.11)	0.521	1 (0.88-1.14)	0.971	0.581
HMDB01348	C18:0 SM	1.06 (0.93-1.21)	0.396	1.03 (0.91-1.17)	0.602	0.973	HMDB01348	C18:0 SM	1.1 (0.96-1.27)	0.176	1.01 (0.89-1.15)	0.844	0.587
HMDB04620	N-alpha-acetylarginine	0.98 (0.85-1.12)	0.726	0.94 (0.83-1.06)	0.285	0.768	HMDB04620	N-alpha-acetylarginine	0.99 (0.86-1.13)	0.842	0.93 (0.82-1.05)	0.248	0.587
HMDB12102	C20:0 SM	1 (0.87-1.16)	0.986	1.04 (0.93-1.18)	0.478	0.536	HMDB12102	C20:0 SM	1 (0.86-1.16)	0.988	1.03 (0.91-1.17)	0.619	0.587
HMDB11211*	C34:3 PC plasmalogen	1.13 (0.99-1.29)	0.08	1.06 (0.93-1.2)	0.381	0.457	HMDB11211*	C34:3 PC plasmalogen	1.08 (0.94-1.25)	0.279	1.07 (0.94-1.21)	0.328	0.61
HMDB10407*	C16:1 LPC plasmalogen	1.08 (0.94-1.24)	0.276	1.03 (0.92-1.16)	0.612	0.653	HMDB10407*	C16:1 LPC plasmalogen	1.06 (0.92-1.22)	0.407	1.03 (0.91-1.16)	0.664	0.617
HMDB00259	serotonin	1.07 (0.94-1.22)	0.296	1.03 (0.91-1.16)	0.68	0.56	HMDB00259	serotonin	1.08 (0.95-1.24)	0.239	1.02 (0.9-1.16)	0.705	0.638
HMDB01431	pyridoxamine	0.88 (0.76-1.02)	0.099	1 (0.88-1.14)	0.994	0.16	HMDB01431	pyridoxamine	0.93 (0.8-1.09)	0.383	0.97 (0.85-1.1)	0.628	0.647
HMDB13288	C9 carnitine	0.99 (0.86-1.14)	0.895	1.01 (0.9-1.13)	0.853	0.716	HMDB13288	C9 carnitine	0.96 (0.83-1.11)	0.566	1.01 (0.9-1.13)	0.865	0.655
HMDB00699	1-methylnicotinamide	0.96 (0.83-1.1)	0.537	1.03 (0.91-1.15)	0.674	0.482	HMDB00699	1-methylnicotinamide	0.93 (0.8-1.08)	0.332	1 (0.89-1.13)	0.995	0.659
HMDB04824	N2,N2-dimethylguanosine	0.88 (0.76-1.01)	0.07	0.98 (0.87-1.1)	0.691	0.344	HMDB04824	N2,N2-dimethylguanosine	0.93 (0.8-1.08)	0.317	0.97 (0.86-1.1)	0.671	0.679
HMDB00206	N6-acetyllysine	0.96 (0.84-1.1)	0.554	0.94 (0.83-1.05)	0.282	0.852	HMDB00206	N6-acetyllysine	0.98 (0.85-1.12)	0.74	0.95 (0.84-1.07)	0.359	0.68
HMDB29416	NMMA	1 (0.86-1.15)	0.951	0.99 (0.87-1.13)	0.919	0.888	HMDB29416	NMMA	1.04 (0.89-1.21)	0.607	0.97 (0.85-1.11)	0.687	0.686
HMDB11517	C20:4 LPE	1.01 (0.88-1.17)	0.882	1.04 (0.93-1.17)	0.494	0.659	HMDB11517	C20:4 LPE	1.02 (0.88-1.18)	0.829	1.05 (0.94-1.19)	0.382	0.702
HMDB00043	betaine	0.99 (0.86-1.14)	0.883	1.03 (0.91-1.17)	0.636	0.708	HMDB00043	betaine	1 (0.87-1.16)	0.997	1.03 (0.91-1.18)	0.613	0.704
HMDB03681	4-acetamidobutanoate	0.96 (0.83-1.1)	0.539	0.96 (0.85-1.08)	0.503	0.95	HMDB03681	4-acetamidobutanoate	0.97 (0.83-1.12)	0.645	0.94 (0.84-1.07)	0.359	0.711
HMDB59824	4-hydroxy-3-methylacetophenone	0.99 (0.87-1.12)	0.821	0.96 (0.85-1.09)	0.555	0.813	HMDB59824	4-hydroxy-3-methylacetophenone	1 (0.87-1.14)	0.961	0.96 (0.85-1.08)	0.508	0.712
HMDB02250	C12 carnitine	0.99 (0.85-1.15)	0.896	1 (0.9-1.13)	0.937	0.587	HMDB02250	C12 carnitine	0.98 (0.84-1.15)	0.813	1 (0.89-1.13)	0.96	0.722
HMDB00897	7-methylguanine	0.98 (0.85-1.12)	0.751	0.98 (0.87-1.09)	0.709	0.985	HMDB00897	7-methylguanine	1.01 (0.88-1.17)	0.873	0.97 (0.86-1.08)	0.559	0.731
HMDB00026	N-carbamoyl-beta-alanine	0.97 (0.85-1.11)	0.675	0.97 (0.86-1.09)	0.6	0.91	HMDB00026	N-carbamoyl-beta-alanine	0.97 (0.85-1.12)	0.707	0.97 (0.86-1.09)	0.582	0.731
NA	C46:3 TAG	0.94 (0.81-1.07)	0.345	0.94 (0.83-1.06)	0.297	0.628	NA	C46:3 TAG	0.95 (0.82-1.09)	0.448	0.94 (0.83-1.06)	0.293	0.746
HMDB00462	allantoin	0.94 (0.82-1.08)	0.387	0.95 (0.84-1.08)	0.439	0.765	HMDB00462	allantoin	1 (0.87-1.16)	0.987	0.95 (0.84-1.08)	0.473	0.751
HMDB10387*	C18:3 LPC	0.99 (0.87-1.13)	0.927	0.98 (0.87-1.11)	0.779	0.886	HMDB10387*	C18:3 LPC	0.99 (0.87-1.13)	0.887	0.96 (0.85-1.09)	0.552	0.755
HMDB00201	C2 carnitine	1 (0.87-1.15)	0.999	1 (0.89-1.12)	0.965	0.661	HMDB00201	C2 carnitine	1.06 (0.92-1.24)	0.409	0.99 (0.87-1.11)	0.807	0.76
HMDB04953*	C24:1 Ceramide (d18:1)	0.98 (0.87-1.11)	0.794	1.01 (0.89-1.14)	0.883	0.626	HMDB04953*	C24:1 Ceramide (d18:1)	0.98 (0.87-1.12)	0.815	1 (0.89-1.14)	0.942	0.76
HMDB01257	spermidine	1.06 (0.92-1.23)	0.393	1.12 (1-1.25)	0.053	0.781	HMDB01257	spermidine	1.1 (0.95-1.27)	0.218	1.11 (0.99-1.25)	0.067	0.763
HMDB11410*	C36:5 PE plasmalogen	1.03 (0.9-1.18)	0.622	1.04 (0.93-1.17)	0.495	0.908	HMDB11410*	C36:5 PE plasmalogen	1.04 (0.91-1.19)	0.586	1.03 (0.92-1.16)	0.588	0.778

HMDB00158	tyrosine	0.95 (0.82-1.1)	0.507	1.01 (0.9-1.14)	0.896	0.397	HMDB00158	tyrosine	1.02 (0.88-1.2)	0.761	0.98 (0.87-1.11)	0.79	0.783
HMDB00715	kynurenic acid	0.87 (0.76-1)	0.05	0.9 (0.8-1.01)	0.08	0.754	HMDB00715	kynurenic acid	0.87 (0.76-1.01)	0.06	0.88 (0.78-0.99)	0.04	0.788
HMDB15150	sulfamethoxazole	1.05 (0.91-1.2)	0.498	1.02 (0.91-1.15)	0.675	0.732	HMDB15150	sulfamethoxazole	1.05 (0.91-1.21)	0.473	1.02 (0.91-1.14)	0.762	0.791
HMDB07869*	C30:0 PC	0.98 (0.85-1.12)	0.743	1 (0.88-1.12)	0.941	0.678	HMDB07869*	C30:0 PC	0.96 (0.83-1.11)	0.568	0.96 (0.85-1.09)	0.55	0.792
HMDB08925*	C34:0 PE	1.03 (0.9-1.18)	0.651	1 (0.89-1.12)	0.973	0.87	HMDB08925*	C34:0 PE	1.02 (0.89-1.17)	0.808	0.98 (0.87-1.1)	0.745	0.799
HMDB13713	N-acetyltryptophan	0.99 (0.87-1.14)	0.916	0.95 (0.85-1.07)	0.405	0.583	HMDB13713	N-acetyltryptophan	0.95 (0.83-1.1)	0.505	0.94 (0.83-1.05)	0.278	0.8
HMDB11253*	C38:5 PE plasmalogen	1.03 (0.9-1.19)	0.666	1.03 (0.92-1.15)	0.588	0.786	HMDB11253*	C38:5 PE plasmalogen	1.04 (0.9-1.2)	0.574	1.04 (0.93-1.17)	0.474	0.808
HMDB10169	C16:0 SM	1.07 (0.94-1.23)	0.314	1.06 (0.94-1.19)	0.356	0.899	HMDB10169	C16:0 SM	1.07 (0.93-1.23)	0.376	1.05 (0.93-1.18)	0.417	0.809
HMDB00210	pantothenate	1.01 (0.88-1.15)	0.938	1.07 (0.95-1.21)	0.265	0.404	HMDB00210	pantothenate	1.03 (0.9-1.19)	0.674	1.04 (0.92-1.18)	0.534	0.811
NA	ectoine	1.02 (0.89-1.16)	0.807	1 (0.9-1.13)	0.937	0.775	NA	ectoine	1.01 (0.88-1.16)	0.868	1 (0.89-1.13)	0.934	0.822
HMDB03282	1-methylguanine	0.97 (0.85-1.11)	0.627	1.03 (0.92-1.15)	0.636	0.611	HMDB03282	1-methylguanine	1 (0.87-1.15)	0.986	1.02 (0.9-1.14)	0.794	0.823
HMDB10384	C18:0 LPC	1.03 (0.9-1.18)	0.635	1.05 (0.93-1.18)	0.454	0.822	HMDB10384	C18:0 LPC	1.03 (0.9-1.18)	0.685	1.05 (0.93-1.18)	0.443	0.826
NA	2-aminohippuric acid	0.85 (0.74-0.98)	0.024	0.87 (0.77-0.97)	0.018	0.945	NA	2-aminohippuric acid	0.86 (0.75-0.99)	0.041	0.87 (0.76-0.98)	0.02	0.828
HMDB06344	phenylacetylglutamine	0.9 (0.79-1.03)	0.127	0.91 (0.81-1.03)	0.132	0.709	HMDB06344	phenylacetylglutamine	0.89 (0.78-1.02)	0.105	0.93 (0.82-1.05)	0.234	0.834
HMDB00168	asparagine	1.12 (0.97-1.29)	0.13	1.1 (0.98-1.25)	0.109	0.91	HMDB00168	asparagine	1.11 (0.95-1.29)	0.183	1.09 (0.97-1.24)	0.159	0.838
HMDB10382	C16:0 LPC	1.01 (0.88-1.15)	0.924	1.04 (0.92-1.17)	0.525	0.628	HMDB10382	C16:0 LPC	1 (0.87-1.14)	0.976	1.03 (0.91-1.16)	0.684	0.842
HMDB10395	C20:4 LPC	1.03 (0.9-1.18)	0.671	1 (0.89-1.12)	0.965	0.672	HMDB10395	C20:4 LPC	1.01 (0.88-1.17)	0.853	1 (0.89-1.13)	0.997	0.845
HMDB00092	dimethylglycine	0.97 (0.85-1.12)	0.712	1.05 (0.94-1.18)	0.393	0.371	HMDB00092	dimethylglycine	1.05 (0.91-1.21)	0.527	1.05 (0.94-1.18)	0.412	0.859
HMDB05015	gabapentin	1 (0.89-1.13)	0.999	1.01 (0.89-1.15)	0.841	0.931	HMDB05015	gabapentin	1.02 (0.9-1.15)	0.792	1 (0.88-1.14)	0.962	0.868
NA	C44:2 TAG	0.95 (0.82-1.09)	0.446	0.92 (0.81-1.03)	0.158	0.933	NA	C44:2 TAG	0.95 (0.82-1.1)	0.52	0.91 (0.8-1.03)	0.137	0.868
HMDB01325	N6,N6,N6-trimethyllysine	0.97 (0.85-1.12)	0.704	0.99 (0.89-1.11)	0.902	0.916	HMDB01325	N6,N6,N6-trimethyllysine	0.97 (0.84-1.11)	0.641	0.98 (0.87-1.1)	0.758	0.873
HMDB11507*	C18:2 LPE	1.03 (0.89-1.19)	0.701	1.02 (0.91-1.15)	0.747	0.905	HMDB11507*	C18:2 LPE	1.02 (0.87-1.18)	0.836	1.03 (0.91-1.16)	0.617	0.877
HMDB11220*	C36:5 PC plasmalogen	1.01 (0.88-1.16)	0.884	1.01 (0.9-1.13)	0.856	0.924	HMDB11220*	C36:5 PC plasmalogen	1 (0.87-1.15)	0.993	1.01 (0.9-1.14)	0.84	0.877
HMDB01855	5-hydroxytryptophol	1.1 (0.97-1.27)	0.149	1.06 (0.94-1.2)	0.315	0.538	HMDB01855	5-hydroxytryptophol	1.07 (0.94-1.24)	0.311	1.06 (0.94-1.19)	0.365	0.888
HMDB03334	SDMA	0.96 (0.83-1.11)	0.58	1.01 (0.9-1.13)	0.889	0.732	HMDB03334	SDMA	0.96 (0.83-1.12)	0.625	1 (0.89-1.12)	0.988	0.889
HMDB00112	GABA	1.01 (0.88-1.16)	0.897	1.05 (0.93-1.19)	0.391	0.643	HMDB00112	GABA	1.04 (0.91-1.2)	0.544	1.04 (0.92-1.18)	0.5	0.892
HMDB00898	1-methylhistamine	1.04 (0.91-1.19)	0.593	1.02 (0.91-1.14)	0.773	0.931	HMDB00898	1-methylhistamine	1.04 (0.91-1.2)	0.542	1.01 (0.9-1.13)	0.877	0.894
Internal Standard	phenylalanine-d8	0.99 (0.84-1.15)	0.859	1.03 (0.92-1.14)	0.634	0.693	Internal Standard	phenylalanine-d8	1.04 (0.88-1.22)	0.654	1.02 (0.92-1.14)	0.716	0.895
NA	C46:2 TAG	0.94 (0.81-1.07)	0.347	0.92 (0.82-1.04)	0.184	0.776	NA	C46:2 TAG	0.94 (0.81-1.09)	0.421	0.92 (0.81-1.04)	0.164	0.906
HMDB01414	putrescine	1.11 (0.97-1.27)	0.124	1.07 (0.95-1.21)	0.279	0.552	HMDB01414	putrescine	1.11 (0.97-1.27)	0.133	1.08 (0.96-1.22)	0.22	0.908
HMDB00853	N-acetylgalactosamine	1.01 (0.88-1.15)	0.917	1.05 (0.93-1.18)	0.43	0.575	HMDB00853	N-acetylgalactosamine	1.1 (0.96-1.27)	0.188	1.05 (0.93-1.18)	0.459	0.911
HMDB01476	3-hydroxyanthranilic acid	1 (0.87-1.14)	0.946	1.02 (0.91-1.14)	0.784	0.859	HMDB01476	3-hydroxyanthranilic acid	0.99 (0.86-1.13)	0.865	1 (0.89-1.12)	0.975	0.913
HMDB07973*	C34:2 PC	0.97 (0.85-1.12)	0.697	1.03 (0.91-1.17)	0.603	0.525	HMDB07973*	C34:2 PC	0.98 (0.85-1.13)	0.741	1.03 (0.91-1.17)	0.654	0.919
HMDB00167	threonine	1.01 (0.87-1.18)	0.868	1.03 (0.91-1.17)	0.652	0.638	HMDB00167	threonine	1.04 (0.89-1.22)	0.637	1.01 (0.88-1.15)	0.911	0.927
HMDB00248	thyroxine	0.99 (0.85-1.14)	0.84	1 (0.89-1.13)	0.934	0.691	HMDB00248	thyroxine	1.01 (0.87-1.18)	0.846	1.01 (0.89-1.13)	0.914	0.928
NA	C36:3 PS plasmalogen	0.98 (0.85-1.14)	0.812	1 (0.9-1.12)	0.975	0.68	NA	C36:3 PS plasmalogen	1 (0.85-1.17)	0.956	0.99 (0.88-1.11)	0.878	0.936
Internal Standard	valine-d8	1 (0.86-1.16)	0.976	1 (0.9-1.11)	0.995	0.962	Internal Standard	valine-d8	1.03 (0.88-1.19)	0.748	1 (0.9-1.12)	0.941	0.938
HMDB02014	C14:1 carnitine	1.01 (0.87-1.17)	0.93	1.01 (0.9-1.13)	0.862	0.694	HMDB02014	C14:1 carnitine	1.02 (0.88-1.2)	0.763	1.01 (0.9-1.13)	0.92	0.938
HMDB00875	trigonelline	0.96 (0.83-1.1)	0.514	0.95 (0.84-1.07)	0.403	0.925	HMDB00875	trigonelline	0.94 (0.81-1.08)	0.369	0.95 (0.84-1.08)	0.428	0.947
HMDB11503	C16:0 LPE	1.04 (0.91-1.19)	0.53	1.05 (0.92-1.19)	0.491	0.802	HMDB11503	C16:0 LPE	1.03 (0.89-1.18)	0.723	1.03 (0.9-1.18)	0.637	0.958
HMDB00767	pseudouridine	0.94 (0.82-1.08)	0.399	0.97 (0.86-1.09)	0.562	0.786	HMDB00767	pseudouridine	0.99 (0.85-1.15)	0.845	0.96 (0.86-1.09)	0.547	0.96
HMDB00925	trimethylamine-N-oxide	1 (0.87-1.15)	0.975	1.02 (0.91-1.14)	0.725	0.734	HMDB00925	trimethylamine-N-oxide	1.03 (0.9-1.19)	0.674	1.03 (0.92-1.16)	0.618	0.962
HMDB00064	creatine	1.07 (0.92-1.23)	0.391	1.13 (1-1.28)	0.046	0.403	HMDB00064	creatine	1.12 (0.96-1.3)	0.152	1.12 (0.99-1.27)	0.076	0.968
HMDB00054	bilirubin	0.95 (0.81-1.11)	0.497	0.95 (0.85-1.06)	0.365	0.911	HMDB00054	bilirubin	0.95 (0.81-1.11)	0.532	0.95 (0.85-1.05)	0.311	0.973
HMDB00719	homoserine	1.01 (0.87-1.18)	0.883	1.04 (0.91-1.18)	0.575	0.59	HMDB00719	homoserine	1.04 (0.88-1.22)	0.668	1.02 (0.89-1.16)	0.823	0.973
HMDB01276	N1-acetylspermidine	1.06 (0.92-1.24)	0.408	1.15 (1.03-1.3)	0.016	0.686	HMDB01276	N1-acetylspermidine	1.13 (0.97-1.32)	0.117	1.15 (1.02-1.3)	0.021	0.976
HMDB02000	myristoleic acid	0.97 (0.84-1.12)	0.651	0.93 (0.83-1.03)	0.169	0.721	HMDB02000	myristoleic acid	0.99 (0.85-1.15)	0.871	0.94 (0.84-1.05)	0.299	0.985
HMDB01539	ADMA	0.96 (0.83-1.11)	0.582	0.99 (0.88-1.1)	0.802	0.685	HMDB01539	ADMA	1.01 (0.87-1.17)	0.938	0.99 (0.88-1.11)	0.888	0.989
HMDB00705	C6 carnitine	0.96 (0.83-1.12)	0.639	1 (0.89-1.13)	0.989	0.468	HMDB00705	C6 carnitine	1 (0.86-1.17)	0.994	0.98 (0.87-1.11)	0.792	0.992
NA	C20:4 carnitine	1.04 (0.91-1.2)	0.558	1 (0.9-1.13)	0.943	0.645	NA	C20:4 carnitine	1.05 (0.91-1.21)	0.492	1.02 (0.91-1.14)	0.755	0.999
