# Supplementary material for: Circulating amino acids and amino acid-related metabolites and risk of breast cancer among predominantly premenopausal women
Source: NPJ Breast Cancer. 2021 May 18;7:54. doi: 10.1038/s41523-021-00262-4 (PMC8131633; doi:10.1038/s41523-021-00262-4)
Supplement: Supplementary file 2 — Reporting Summary [file 41523_2021_262_MOESM2_ESM.pdf]

nature research

Corresponding author(s): Oana A. Zeleznik

Last updated by author(s): Apr 12, 2021

Reporting Summary

Nature Research wishes to improve the reproducibility of the work that we publish. This form provides structure for consistency and transparency in reporting. For further information on Nature Research policies, see our Editorial Policies and the Editorial Policy Checklist.

Statistics

For all statistical analyses, confirm that the following items are present in the figure legend, table legend, main text, or Methods section.

n/a	Confirmed	
☐	☒	The exact sample size (*n*) for each experimental group/condition, given as a discrete number and unit of measurement
☐	☒	A statement on whether measurements were taken from distinct samples or whether the same sample was measured repeatedly
☐	☒	The statistical test(s) used AND whether they are one- or two-sided<br>*Only common tests should be described solely by name; describe more complex techniques in the Methods section.*
☐	☒	A description of all covariates tested
☐	☒	A description of any assumptions or corrections, such as tests of normality and adjustment for multiple comparisons
☐	☒	A full description of the statistical parameters including central tendency (e.g. means) or other basic estimates (e.g. regression coefficient) AND variation (e.g. standard deviation) or associated estimates of uncertainty (e.g. confidence intervals)
☐	☒	For null hypothesis testing, the test statistic (e.g. *F*, *t*, *r*) with confidence intervals, effect sizes, degrees of freedom and *P* value noted<br>*Give P values as exact values whenever suitable.*
☒	☐	For Bayesian analysis, information on the choice of priors and Markov chain Monte Carlo settings
☒	☐	For hierarchical and complex designs, identification of the appropriate level for tests and full reporting of outcomes
☐	☒	Estimates of effect sizes (e.g. Cohen's *d*, Pearson's *r*), indicating how they were calculated

Our web collection on statistics for biologists contains articles on many of the points above.

Software and code

Policy information about availability of computer code

Data collection	Questionnaire data: SAS v 9.4.<br>TraceFinder versions 3.1 and 3.2 (Thermo Fisher, Waltham MA) and Progenesis QI version 1.0.5165.27075 (Nonlinear Dynamics, Durham NC).
Data analysis	R version 3.6.3 and SAS v 9.4 run on CentOS release 6.10; R-packages: Biobase_2.46.0, stats4_3.6.3, clogitL1, randomForest

For manuscripts utilizing custom algorithms or software that are central to the research but not yet described in published literature, software must be made available to editors and reviewers. We strongly encourage code deposition in a community repository (e.g. GitHub). See the Nature Research guidelines for submitting code & software for further information.

Data

Policy information about availability of data

All manuscripts must include a data availability statement. This statement should provide the following information, where applicable:

- Accession codes, unique identifiers, or web links for publicly available datasets
- A list of figures that have associated raw data
- A description of any restrictions on data availability

Data cannot be shared publicly because data access must be approved by the institutional review boards of the Brigham and Women's Hospital and Harvard T.H. Chan School of Public Health. Inquiries regarding data access are encouraged through http://www.nurseshealthstudy.org/researchers. Access to statistical code and datasets will be facilitated following the existing data sharing guidelines provided, which can be found on the study website.

Field-specific reporting

Please select the one below that is the best fit for your research. If you are not sure, read the appropriate sections before making your selection.

☒ Life sciences ☐ Behavioural & social sciences ☐ Ecological, evolutionary & environmental sciences

For a reference copy of the document with all sections, see nature.com/documents/nr-reporting-summary-flat.pdf

Life sciences study design

All studies must disclose on these points even when the disclosure is negative.

Sample size	Available funding to measure metabolomics
Data exclusions	No data were excluded
Replication	We do not have access to a similar dataset (prospective breast cancer study of metabolomics among premenopausal women) to perform a replication study.
Randomization	Cases of breast cancer were identified after blood collection among women who had no reported cancer (other than nonmelanoma skin). 1057 cases (invasive cases n=780) were diagnosed between 1999 and 2011. Breast cancer cases were reported by the participant, which were confirmed by medical record reviews (n=1015) or verbally by the nurse (n=42). Given the high confirmation rate by medical record for breast cancer in this cohort (99%), all cases are included in this analysis.<br>One control was matched per case by the following factors: age (+/- 2y), menopausal status and postmenopausal hormone therapy (HT) use at blood collection and diagnosis (premenopausal, postmenopausal and not taking HT, postmenopausal and taking HT, and unknown), and month (+/- 1mo), time of day (+/- 2h), fasting status at blood collection (<8 h after a meal or unknown; >10h), race/ethnicity (African-American, Asian, Hispanic, Caucasian, other) and luteal day (+/- 1d; timed samples only).
Blinding	Investigators at the Broad Institute who measured metabolomics data were blinded to the case-control status of the study participants.

Reporting for specific materials, systems and methods

We require information from authors about some types of materials, experimental systems and methods used in many studies. Here, indicate whether each material, system or method listed is relevant to your study. If you are not sure if a list item applies to your research, read the appropriate section before selecting a response.

Materials & experimental systems

n/a	Involved in the study	
☒	☐	Antibodies
☒	☐	Eukaryotic cell lines
☒	☐	Palaeontology and archaeology
☒	☐	Animals and other organisms
☐	☒	Human research participants
☒	☐	Clinical data
☒	☐	Dual use research of concern

Methods

n/a	Involved in the study	
☒	☐	ChIP-seq
☒	☐	Flow cytometry
☒	☐	MRI-based neuroimaging

Human research participants

Policy information about studies involving human research participants

Population characteristics	1057 cases and 1057 matched controls were included in this study. Women were an average 53 years old and predominantly premenopausal (80%) at the time of blood collection. At diagnosis, 42% of the women were premenopausal and 46% were postmenopausal. The mean time between blood collection and diagnosis was 8 years (SD=4.4), ranging from 10 months to 17.4 years. (1st quartile: 4.25 years; 3rd quartile: 11.6 years). Additional charactersitics are presented in Table 1.
Recruitment	Our cohort consisted of registered nurses, a group that are not representative of the general population (e.g. social economic status), however there is no evidence suggesting that breast carcinogenesis is different in this group of women. Additionally, our cohort also included predominantly Caucasian women. However, while the prevalence of risk factors often differs across population subgroups, many breast cancer risk factors have been documented to operate similarly across ethnic groups, as would be expected from a common underlying biology. Nonetheless, it is crucial that future studies conduct similar analyses in racially and ethnically diverse cohorts.
Ethics oversight	The study protocol was approved by the institutional review boards of the Brigham and Women's Hospital and Harvard T.H. Chan School of Public Health, and those of participating registries as required.

Note that full information on the approval of the study protocol must also be provided in the manuscript.
